# Regioselective and enantioselective propargylic hydroxylations catalyzed by P450tol monooxygenases

**DOI:** 10.1186/s40643-024-00771-7

**Published:** 2024-07-02

**Authors:** Xu Deng, Cheng-Cheng Song, Wen-Jing Gu, Yu-Jie Wang, Lu Feng, Xiao-Jian Zhou, Ming-Qiang Zhou, Wei-Cheng Yuan, Yong-Zheng Chen

**Affiliations:** 1 Key Laboratory of Biocatalysis & Chiral Drug Synthesis of Guizhou Province, Key Laboratory of Basic Pharmacology of Ministry of Education, School of Pharmacy, Zunyi Medical University, Zunyi, China; 2 National Engineering Research Center of Chiral Drugs, Chengdu Institute of Organic Chemistry, Chinese Academy of Sciences, Chengdu, 610041 China

**Keywords:** Biocatalysis, Hydroxylation, P450 monooxygenase, Propargylic alcohols, Enantioselectivity

## Abstract

Regioselective and enantioselective hydroxylation of propargylic C-H bonds are useful reactions but often lack appropriate catalysts. Here a green and efficient asymmetric hydroxylation of primary and secondary C–H bonds at propargylic positions has been established. A series of optically active propargylic alcohols were prepared with high regio- and enantioselectivity (up to 99% *ee*) under mild reaction conditions by using P450tol, while the C≡C bonds in the molecule remained unreacted. This protocol provides a green and practical method for constructing enantiomerically chiral propargylic alcohols. In addition, we also demonstrated that the biohydroxylation strategy was able to scaled up to 2.25 mmol scale with the production of chiral propargyl alcohol **2a** at a yield of 196 mg with 96% *ee*, which’s an important synthetic intermediate of antifungal drug Ravuconazole.

**Graphical Abstract:**

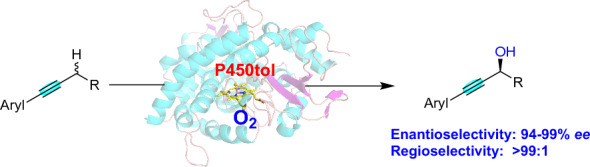

**Supplementary Information:**

The online version contains supplementary material available at 10.1186/s40643-024-00771-7.

## Introduction

Chiral propargylic alcohols are useful and versatile motifs that can be transformed into chiral allylic alcohols, allenes, bioactive molecules and natural products (Bauer 2012; Greshock et al. 2008; Helal et al. 1996; Lumbroso et al. 2013; Nakayama et al. 2011; Wang and Pu 2013). Over the past few decades, there are three mainly standard synthetic methods for the preparation of enantiopure propargylic alcohols: (1) the asymmetric transfer hydrogenation of alkynyl ketones catalyzed by transition metal (Matsumura et al. 1997; Shatskiy et al. 2015; Zhang et al. 2018); (2) the asymmetric addition of alkynyl organometallic reagents to aldehydes or ketones (Corey and Cimprich 1994; Lu et al. 2005; Trost and Weiss 2009); (3) the deracemization of racemic propargylic alcohols catalyzed by biocatalytic reaction (González-Granda et al. 2020; Kawanishi et al. 2019; Sang et al. 2022; Saravanan et al. 2014). Although some of these strategies access to chiral propargylic alcohols have been developed, many of them involve the use of transition metal catalyst, air sensitive reagents, or deliver chiral propargylic alcohols with moderate enantioselectivity. Up to date, it is still a challenge to develop a biocatalytic method with monooxygenase for the synthesis of enantiopure propargylic alcohols.

As we all known, enantioselective oxidation of propargylic C-H bond is the most direct and atom economic strategy for the preparation of enantiopure propargylic alcohols. Until now, only two examples have been reported to prepare chiral propargylic alcohols: (1) Cu(MeCN)_4_PF_6_ and chiral bisoxazoline ligand catalyzed acyloxylation of propargylic C-H bond to obtained the products with 15–51% *ee* under an excess of oxidant and reactions took 4–5 days to proceed (Stephen Clark et al. 1998); (2) Hydroxylation of propargylic C-H Bond catalyzed by Chloroperoxidase (CPO) using equivalent of H_2_O_2_ or TBHP as terminal oxidant to synthesize propargylic alcohols with 57–95% *ee* or 43–90% *ee*, respectively (Hu and Hager 1999). However, selective C-H oxidation of simple alkynes represents one of the most fundamental challenges. On the one hand, the highly active compound I species are usually undistinguishing toward similar C-H bonds, resulting in low chemoselectivity (overoxidation products) (Alvarez et al. 2007; Hu and Hager 1999) and regioselectivity, On the other hand, alkynyl and alkyl groups with relatively small steric effect are difficult to differentiate by small molecular catalysts, resulting in low stereoselectivity (Stephen Clark et al. 1998). Further development of higher stereoselectivity and more efficient catalysts in this field is therefore of great significance for asymmetric catalysis.

**Fig. 1:**
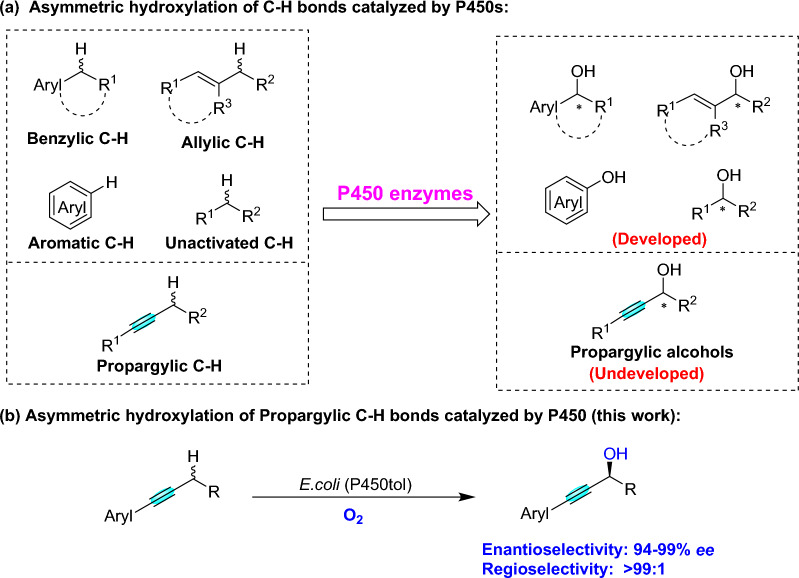
Asymmetric hydroxylation of C-H bonds catalyzed by P450s

Cytochrome P450 monooxygenases (P450s) catalyzed the oxidative reactions of C-H bonds with stereo- and regioselective manner under mild reaction condition (Chakrabarty et al. 2020; Chen et al. 2023; Jiang et al. 2021; Li et al. 2020; Li and Wong 2019; Manning et al. 2019; Roiban and Reetz 2015; Song et al. 2023; Whitehouse et al. 2012; Zhang et al. 2022a, 2023, 2022b) Additionally, P450s as mild and selective catalysts have been used in the asymmetric hydroxylation of benzylic, allylic, aromatic and unactivated C-H bonds (Fig. 1a) (Chakrabarty et al. 2020; Kim et al. 2022; Neufeld et al. 2014; Roiban and Reetz 2015; Whitehouse et al. 2012). However, P450s as an effective and versatile catalyst for the enantioselective hydroxylation of propargylic C-H bonds has not been available in literature. In light of our ongoing interests in P450s-catalyzed asymmetric hydroxylation reactions with a broad substrate range and widely applications (Cui et al. 2023, 2019; Deng et al. 2022; Wan et al. 2022; Wang et al. 2022; Xie et al. 2020, 2023), we herein describe chiral propargylic alcohols were synthesized from the simple alkynes by P450tol catalyzed asymmetric propargylic C-H bonds hydroxylation with regio- and stereoselectivity (Fig. 1b). Additionally, molecular docking and MD simulations were carried out to provide a rationale for the enantioselectivity and regioselectivity of these reactions. As far as we know, this is the first example of asymmetric hydroxylation of propargylic C-H bonds with excellent regio- and enantioselectivity by P450 monooxygenase, while the C≡C bonds in the molecule remained unreacted.

## Materials and methods

### Materials and procedures

Chemicals were purchased from commercial suppliers and used without further purification unless otherwise stated. Isopropyl-*β*-D-thiogalactopyranoside (IPTG) and Ampicillin Sodium Salt (*Amp*) and Streptomycin sulface (*Str*) were purchased from Solarbio (Beijing, China). Analytical thin layer chromatography (TLC) was performed on precoated silica gel 60 GF254 plates. Flash column chromatography was carried out with 300–400 mesh silica gel. The Alkynes and racemic products were synthesized following the reported method (Liu et al. 2022; Watanabe et al. 2018). Visualization on TLC was achieved by use of UV light (254 nm). ^1^H-NMR (400 MHz) and ^13^C-NMR (100 MHz) were recorded on Agilent Technologies 400 MR. Chemical shifts were reported in parts per million (ppm) relative to residual signals of the solvent. The following abbreviations are used to indicate multiplicity: s = singlet, d = doublet, t = triplet, m = multiplet, dd = doublet of doublets. High-resolution mass spectra (HRMS) was recorded by ESI ionization sources. Chiral HPLC analysis was performed on Shimadzu LC-20A, equipped with Chiralpak^®^ OJ-H, OD-H, AD-H or AS-H columns.

### Enzyme preparation

Cultivation of *E. coli* (P450tol-4) cells was carried out using TB medium containing 50 μg/mL *Str* and *Amp*. After growing at 37 °C to an OD_600_ of 0.6–0.8, IPTG was added to the final concentration of 0.2 mM. The culture was incubated at 25 °C with another 12–14 h for enyzme expression. Recombinant *E. coli* (P450tol-4) cells were harvested by centrifugation at 7000 × g at 4 °C for 5 min. The freshly prepared *E. coli* (P450tol-4) cells were resuspended in reaction buffer solution to a cell density (g cdw/L) for performing biotransformation.

### Molecular docking and molecular dynamics simulation

Docking study of **1f** in the active site of enzyme P450tol (PDB No.: 7V40) was carried out using AutoDock 4.0 software (Trott and Olson 2010). All the docking experiments were performed using “Genetic Algorithm” search parameters and default docking parameters. All structural illustrations were generated using the PyMOL software (Seeliger and de Groot 2010).

Molecular dynamics simulation was carried out for P450tol to further explain the mechanism of regio- and enantioselective hydroxyation of substrate **1f**. The molecule structure of **1f** was docked into the active site of enzyme P450tol. The iron-oxo intermediate involved in the cytochrome catalyzed oxidative hydroxylation cycle was used to model the active form of the P450 cofactor (Narayan et al. 2015). Simulations were performed using the GPU code (pmemd) of the AMBER 22 software package (D.A. Case 2017). The Amber-compatible parameters developed by Cheatham et al. (Shahrokh et al. 2012) were used for Cpd I and its axial Cys ligand. Substrate **1f** parameters for the molecular dynamics (MD) simulations were generated within the antechamber module of AMBER 22 using the general AMBER force field (GAFF) (Wang et al. 2004), with partial charges set to fit the electrostatic potential generated at the HF/6-31G(d) level by the restrained electrostatic potential (RESP) model (Bayly et al. 1993). The charges were calculated according to the Merz-Singh-Kollman scheme (Besler et al. 1990; Singh and Kollman 1984) using Gaussian 16(C.01) (M.J. Frisch 2016). Amino acid protonation states were predicted using the H +  + server (http://biophysics.cs.vt.edu/H + +) (Anandakrishnan et al. 2012). Then, the enzyme was solvated in a pre-equilibrated truncated hexagonal box with a 10-Å buffer of TIP3P (Jorgensen et al. 1983) water molecules using the AMBER 22 leap module, resulting in the addition of ∼19000 solvent molecules. The systems were neutralized by addition of ions Na^+^ and Cl^−^, all subsequent calculations were done using the Amber ff14SB force field (Maier et al. 2015). The following MD simulation steps as reported in ref. (Narayan et al. 2015), three independent replicas of 60 ns production trajectories MD simulations were performed after equilibrated. All the structural images and distance were performed using the VMD Software, trajectory analysis and energy was post-processed by Cpptraj module (Maier et al. 2015), respectively. All the structural images and distance were performed using the VMD Software. Trajectory data, root mean square deviation (RMSD), root mean square fluction (RMSF) were analyzed by Cpptraj module, respectively.

### General procedure for the synthesis of chiral (S)-2 on preparation-scale

To a 500-mL shake flask containing a resting cell suspension of *E. coli* (P450tol-4) (10 g cdw/L) and **1** (0.6 mmol) in 180 mL PB buffer (50 mM, pH 8.5). The reaction mixture was shaken at 20 ℃ for 6 h. Then the mixture was extracted with ethyl acetate (3 × 180 mL). The organic phases were separated by centrifugation (7000 × g, 15 min), combined, dried over anhydrous Na_2_SO_4_, and evaporated at reduced pressure. The resulting mixture was purified by flash chromatography using ethyl acetate/petroleum ether as eluent on silica gel to afford the desired chiral product **2**.

### Procedure for the synthesis of chiral (S)-2a on 2.25 mmol-scale

To a 2.0-L shake flask containing a resting cell suspension of *E. coli* (P450tol-4) (10 g cdw/L) and **1** (2.25 mmol) in 750 mL PB buffer (50 mM, pH 8.5). The reaction mixture was shaken at 20 ℃ for 6 h. Then the mixture was extracted with ethyl acetate (3 × 1000 mL). The organic phases were separated by centrifugation (7000 × g, 15 min), combined, dried over anhydrous Na_2_SO_4_, and evaporated at reduced pressure. The resulting mixture was purified by flash chromatography using ethyl acetate/petroleum ether as eluent on silica gel to afford the desired chiral product **2a** (58% yield, 196 mg).

## Results and discussion

### Screening of reaction conditions for propargylic hydroxylation

In our previous work, we have obtained several cytochrome P450 monooxygenases (P450DA, P450PL2-2) (Cui et al. 2023, 2019; Deng et al. 2022; Wan et al. 2022; Wang et al. 2022; Xie et al. 2020, 2023) and also constructed five recombinant *Escherichia coli* strains (P450tol-1 to P450tol-5) harboring P450tol monooxygenase from *Rhodococcus coprophilus* TC-2 (Chen et al. 2022; Li et al. 2014) and five pairs of redox partner Fdx-FdR (ferredoxin-ferredoxin reductase) from *P. lavamentivorans* DS-1 (Wu et al. 2018). With these P450s in hand, but-1-yn-1-ylbenzene (**1a**) was used as a model substrate to explore the hydroxylation activity and stereoselectivity of these recombinant P450 strains. Biohydroxylation reactions were carried out using *E. coli* whole-cells as catalyst without additional cofactors required. After incubation at 30 °C in PB buffer (pH = 8.0) for 6 h, the *ee* and yield of the chiral product 4-phenylbut-3-yn-2-ol (**2a**) were analyzed using chiral HPLC (Table 1). Strains P450DA and P450PL2-2 exhibited the opposite stereoselectivity despite a very low yield in the propargylic C-H hydroxylation reactions, which produced **2a** in 70% *ee* for *R* stereoselectivity and 36% *ee* for *S* stereoselectivity, respectively (Table 1, entries 1–2). Surprisingly, the five recombinant *E. coli* P450tol strains could convert **1a** to **2a** with 96% *ee* and *S* stereoselectivity despite in different yields (Table 1, entries 3–7), where the strain P450tol-4 showed the highest yield of 58% (Table 1, entry 6).

**Table 1:** Screening of P450 strains for asymmetric hydroxylation of **1a**


Entry^*a*^	Biocatalysts	Yield 2a (%)^*b*^	Yield 3a (%)^*b*^	*ee* 2a (%)^*b*^
1	P450DA	3	n.d.^*c*^	70 (*R*)
2	P450PL2-2	1	n.d	36 (*S*)
3	P450tol-1	46	n.d	96 (*S*)
4	P450tol-2	49	n.d	96 (*S*)
5	P450tol-3	48	n.d	96 (*S*)
6	P450tol-4	58	n.d	96 (*S*)
7	P450tol-5	41	n.d	96 (*S*)

^*a*^The reaction was carried out on an analytical scale in 5 mL PB buffer (50 mM, pH = 8.0) containing 10 g cdw/L *E. coli* (P450s) cells with **1a** (2 mM) at 30 °C for 6 h

^*b*^The yield and *ee* were measured by chiral HPLC analysis, and absolute configuration was confirmed by previously reported references (Shatskiy et al. 2015; Watanabe et al. 2018; Zhang et al. 2018).

^*c*^Not detected.

**Fig. 2:**
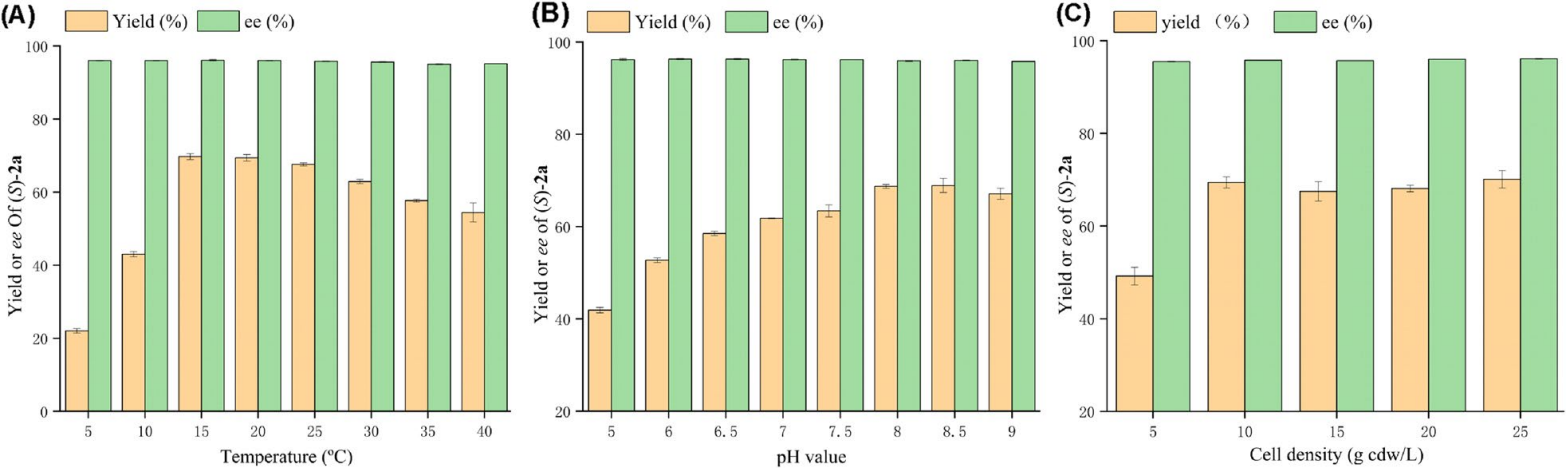
Conditions optimization for asymmetric hydroxylation of **1a** catalyzed by P450tol-4. **A** reaction temperature; **B** reaction pH; **C** cell density

With the optimal strain P450tol-4 in hand, the reaction conditions including temperature, pH of the buffer and cell density of biocatalyst were evaluated. The results are summarized in Fig. 2. From these results, we can find that the stereoselectivity of the reaction remains unchanged with different temperature, pH and cell concentration. With the increase of temperature from 5 to 15 °C, the yield increased from 22 to 69%, while the yield decreased from 69 to 54% with the increase of temperature from 20 to 40 °C. The yields and *ee* values of reactions at 15 and 20 °C are similar, and we choose 20 °C as the reaction temperature for subsequent studies (Fig. 2A). The yield of **2a** improved with the increasing of pH from 5.0 to 8.5, while a higher yield was not obtained by further increasing pH to 9.0 (Fig. 2B). The results in Fig. 2C indicated that the cell density of P450tol influenced the yield of **2a** slightly. The yield of **2a** increased with the increasing of cell concentration from 5 to 10 g cdw/L, while a higher yield was not obtained by further increasing cell density from 10 to 25 g cdw/L. To sum up, the optimal reaction conditions of propargylic hydroxylations were set at 20 °C and reaction pH = 8.5 using 10 g cdw/L cell density of recombinant *E. coli* cells (P450tol-4).

### Scope of substrates

**Fig. 3:**
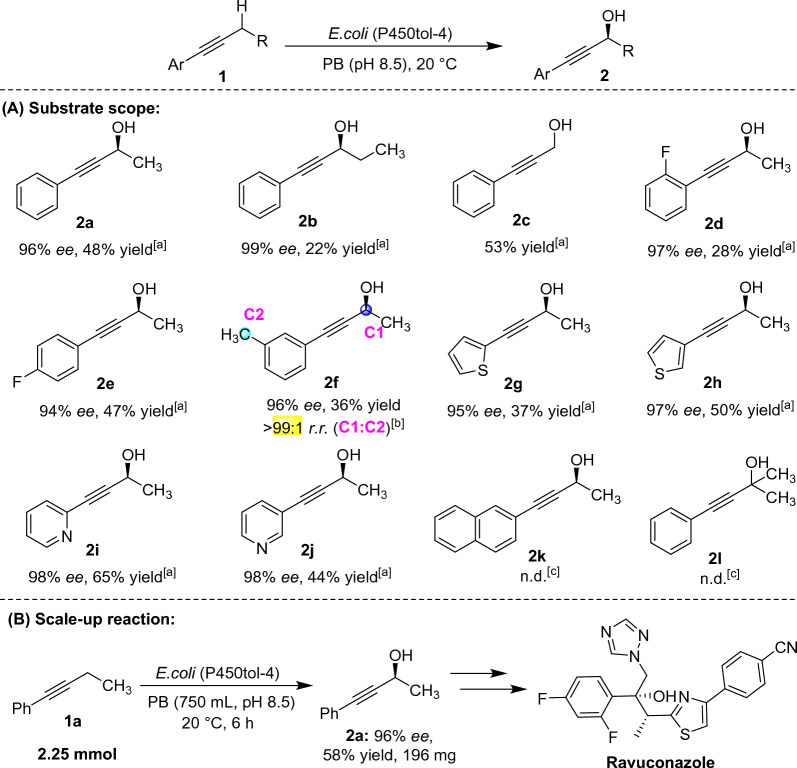
Substrate scope and Scale-up reaction. **A** Substrate scope of *E. coli* (P450tol-4)-catalyzed hydroxylation of alkynes **1**. General conditions: the reaction was carried out on preparation-scale in 180 mL PB buffer (50 mM, pH = 8.5) containing 10 g cdw/L *E. coli* (P450tol-4) cells with **1a** (3 mM) at 20 °C for 6 h.^[a]^ The *ee* values were determined by chiral HPLC, and the isolated yields were obtained by silica gel chromatography.^[b]^ The regioselectivity ratio (**C1:C2**) of propargylic and benzylic C-H bonds hydroxylation was determined by HPLC.^[c]^Not detected. **B** Scale-up reaction of *E. coli* (P450tol-4)-catalyzed hydroxylation of alkyne **1a**

**Fig. 4:**
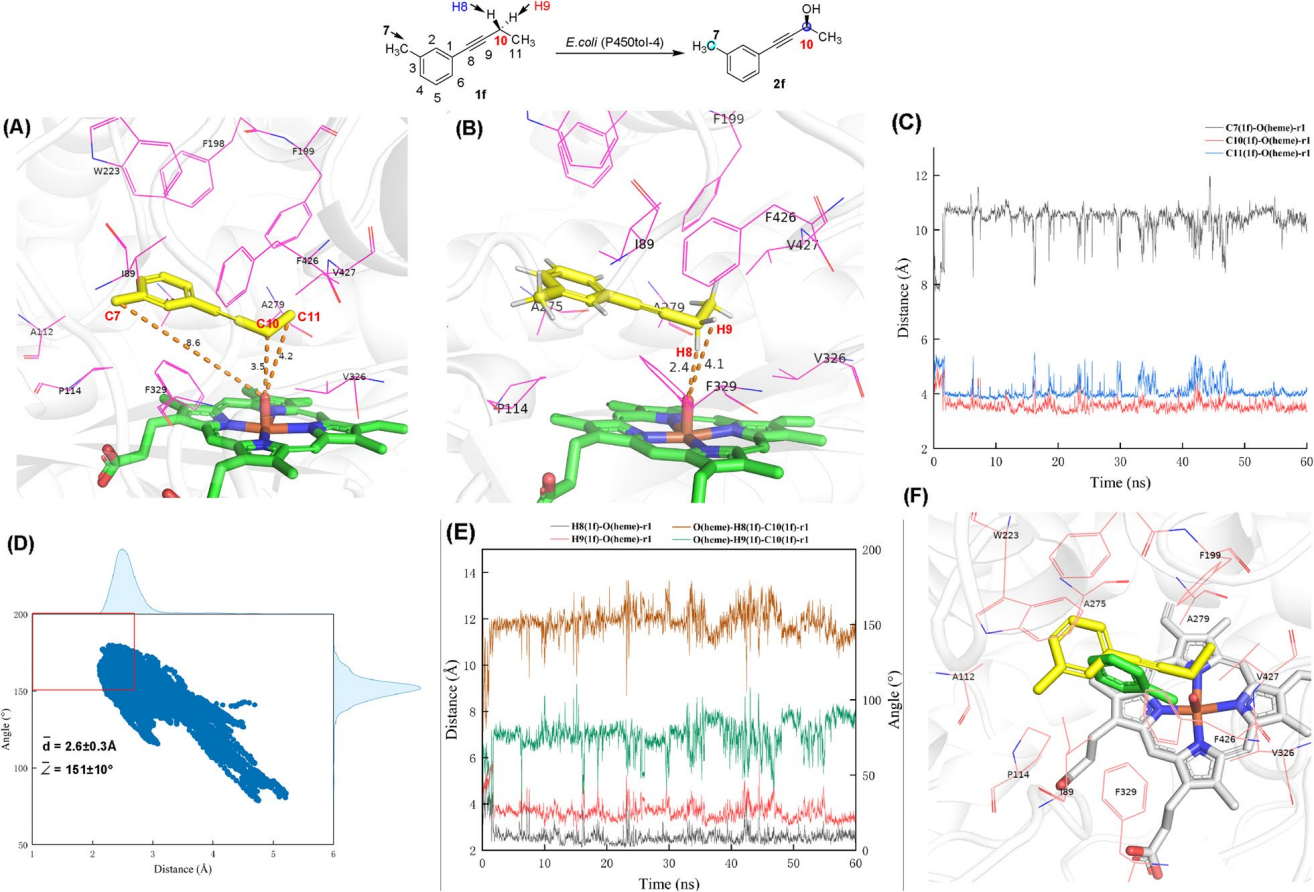
Molecular docking and molecular dynamics simulation (residues labeled in red lines, the distance marked in orange dashed lines). **A**, **B** The docked conformation of substrate **1f** (marked in yellow stick) in the active pocket of P450tol (PDB No.: 7V40); **C** The fluctuation of the distance of the C7(**1f**)-O(heme), C10(**1f**)-O(heme) and C11(**1f**)-O(heme) during the MD simulations; **D** Distances determined between the O(heme) and H8(**1f**) (x axis) and angles formed by O(heme) − H8(**1f**) − C10(**1f**) (y axis) during the MD simulations (3 joint MD replicas). The red box indicates satisfactory conditions of active poses showing both the distance (H8(**1f**)-O(heme)) ≤ 2.7 Å and the angle (O(heme) − H8(**1f**) − C10(**1f**)) ≥ 150°. **E** The fluctuation of the H8(**1f**)-O(heme) and H9(**1f**)-O(heme) distances (y primary axis) and the O(heme)-H8(**1f**)-C10(**1f**) and O(heme)-H9(**1f**)-C10(**1f**) angles (y secondary axis) along the simulation time (x axis) for one of the replicas ( see Figure S2 for replicas 2 and 3); **F** Comparison of substrate binding orientations of the toluene structure (green) with the **1f** bound structure (yellow) based on the P450-toluene complex crystal (PDB: 7V41)

Under the optimal reaction conditions, a range of aryl alkyne substrates **1** were transformed into propargylic alcohols **2** on preparative-scale, and the results are displayed in Fig. 3A. Alkyne containing longer alkyl chain could be converted to the corresponding propargyl alcohol **2b** by P450tol-4 with 99% *ee*, albeit in lower isolated yield (22% yield). Besides hydroxylation of secondary C(*sp*^3^)-H bonds, P450tol-4 could also catalyze the hydroxylation reaction of primary C − H bond in moderate isolated yield (**2c**). Furthermore, substrates with fluorine substitution at *ortho*-, and *para*-positions of the aromatic ring (**2d** and **2e**) were well tolerated in this reaction in 28–47% yield with 94–97% *ee*. In the previous reported work, P450tol monooxygenase catalyzed the benzylic C-H bonds hydroxylation of toluenes to produce benzyl alcohols (Chen et al. 2022). When a methyl substitution in the aromatic ring, there is regioselectivity competition between the benzylic and propargylic C-H bonds in the hydroxylation reaction. The methyl substitution at *meta*-position of the aromatic ring, the product propargyl alcohol **2f** was obtained with 99:1 regioselectivity ratio (*r.r.*) and 96% *ee*. Alkynes containing various heteroaromatic rings, such as furan and pyridine, were transformed to propargylic alcohols **2g**-**2j** as well with 95–98% *ee* in moderate isolated yield. Unfortunately, P450tol-4 exhibited no catalytic efficiency of alkynes containing the bulky naphthalene ring (**2 k**) and tertiary propargylic C–H bond (**2 l**). The results indicating that P450tol preferred the propargylic C-H in **1c** and C7-H in **1f** (Fig. 4) over the terminal unactivated C11-H bonds in **1f** due to the bond dissociation energy of propargylic C-H is lower than that of terminal unactivated C-H bonds. To show the synthetic potential of this strategy, our catalytic reaction can be scaled up to 2.25 mmol scale for the formation of propargyl alcohol **2a** in 58% isolated yield (196 mg) with 96% *ee* (Fig. 3B). In addition, chiral propargyl alcohol **2a** is the important building block for synthesis of antifungal drug Ravuconazole (Xu et al. 2009).

### Molecular docking and molecular dynamics simulation

To obtain a structure-based understanding of the excellent enantio- and regioselectivity of these reactions, molecular docking of the substrate **1f** onto the X-ray structure of P450tol (PDB No.: 7V40) was carried out (Fig. 4A). In the **1f**-enzyme binding pose, the distance between C10-carbon (propargylic-position), C11-carbon (terminal-position) or C7-carbon (benzylic-position) of **1f** and heme oxygen atom (heme-O) of P450tol is 3.5, 4.2 or 8.6 Å, respectively (Fig. 4A). Meanwhile, the distance between C10 pro-*S*-hydrogen or pro-*R*-hydrogen of **1f** and heme-O is 2.4 Å or 4.1 Å, respectively (Fig. 4B). Further MD simulations indicated that such binding conformations of substrate **1f** are stable during 60 ns-MD simulation, during which the mean distance between the C10, C11 or C7 of **1f** and the heme-O was 3.6 ± 0.3, 4.1 ± 0.3 or 10.3 ± 0.5 Å, respectively (Fig. 4C), indicating that **1f** binding in pose C10 is more stable, which is consistent with the selectivity data (> 99:1 regioselectivity at C10-position). Another important finding from our MD simulation study is that the C10 pro-*S*-hydrogen (H8) of **1f** is well positioned for H-abstraction than C10 pro-*R*-hydrogen (H9) with the mean distance of H8(**1f**)-O(heme) of 2.6 ± 0.3 Å and the mean angle of O(heme)-H8(**1f**)-C10(**1f**) of 151 ± 10°, which is consistent with the selectivity data (96% *ee* for *S*-selectivity) (Fig. 4D, E). Furthermore, we refered to the P450-toluene complex crystal (PDB: 7V41) reported by Li (Chen et al. 2022), in which the toluene is located in a hydrophobic pocket and surrounded by I89, A112, P114, F198, F199, W223, A275, A279, F329, F426, and V427. We then overlaid the toluene-bound P450tol structure (PDB: 7V41) with the **1f**-P450tol docking model structure and compared the substrate binding orientations of the toluene structure (green) with the **1f** structure (yellow). We find that the binding orientations of two substrates are similar (Fig. 4F), where the C≡C bonds of **1f** and the toluene phenyl ring were clamped by the residues F329 and A279, and the C10 of **1f** and methyl group of the toluene closed to heme oxygen atom, suggesting that the hydroxylation reaction occurs at the C10 position due to hydrophobic interaction between substrate **1f** and the binding pocket of P450tol. In summary, our MD simulation results provide valuable molecular insights into the excellent enantio- and regioselectivity hydroxylation of C-H bonds in alkynes catalyzed by P450tol.

## Conclusions

In conclusion, we have developed a green and straightforward platform for the asymmetric hydroxylation of primary and secondary C–H bonds at propargylic positions catalyzed by P450tol monooxygenase, while the C≡C bonds in the molecule remained unreacted. This protocol provides a practical and sustainable method for the preparation of enantiomerically pure propargylic alcohols with high regioselectivity (> 99:1 *r.r.*) and enantioselectivity (94–99% *ee*), which are valuable and versatile synthetic building blocks in organic synthesis. Additionally, molecular docking and MD simulations were performed to provide a rationale for the excellent enantio- and regioselectivity of these reactions. Efforts broadening the substrate specificity (e.g., for **1 k** and **1 l**) and inverting the enantioselectivity of the propargylic C-H bonds hydroxylation via engineered P450tol variants are currently ongoing in our laboratory.

### Supplementary Information

Supplementary material 1: Additional Tables S1–S3, Characterization data for the products of chiral (*S*)-**2a**, Amino acid and DNA sequences of P450tol, and HPLC and NMR spectra.

## Data Availability

All data generated or analyzed during this study are included in this article.

## Abbreviations

HPLC: High Performance Liquid chromatography
P450tol: P450 monooxygenase from *Rhodococcus* *coprophilus* TC-2
P450DA: P450 monooxygenase from *Deinococcus* *apachensis*
P450PL2: P450 monooxygenase from *P.* *lavamentivorans* DS-1
Fdx-FdR: Five pairs of redox partner Fdx-FdR (ferredoxin-ferredoxin reductase) from *P.* *lavamentivorans* DS-1
PB buffer: Consist of sodium phosphate dibasic and potassium phosphate monobasic
MD: Molecular dynamics simulation
