# Supplementary material for: Regioselective and enantioselective propargylic hydroxylations catalyzed by P450tol monooxygenases
Source: Bioresour Bioprocess. 2024 Jul 2;11(1):64. doi: 10.1186/s40643-024-00771-7 (PMC11219674; doi:10.1186/s40643-024-00771-7)

**Supporting Information**

**Regioselective and enantioselective propargylic hydroxylations catalyzed by P450tol monooxygenases**

Xu Deng^1^,**^†^**, Cheng-Cheng Song^1^,**^†^**, Wen-Jing Gu^1^,**^†^**, Yu-Jie Wang^1^, Lu Feng^1^, Xiao-Jian Zhou^1,*^, Ming-Qiang Zhou^2^, Wei-Cheng Yuan^2^, Yong-Zheng Chen^1,*^

*^a^ Key Laboratory of Biocatalysis & Chiral Drug Synthesis of Guizhou Province, Key Laboratory of Basic Pharmacology of Ministry of Education, School of Pharmacy, Zunyi Medical University, Zunyi, China.*

*^b^* *National Engineering Research Center of Chiral Drugs, Chengdu Institute of Organic Chemistry, Chinese Academy of Sciences, Chengdu, 610041, China*

*^†^ Xu Deng, Cheng-Cheng Song and Wen-Jing Gu contributed equally to this report.*

**Contents**

1. Supporting Tables S1 to S3......................................................................................2
2. Supporting Figure S1.to S2......................................................................................3

3. Characterization data for the products of chiral (*S*)-**2a**……………..………..……4

4. Amino acid and DNA sequences of P450tol............................................................8

5. HPLC spectra.........................................................................................................10

6. NMR spectra..........................................................................................................19

**1. Tables S1 to S3**

**Tables S1** Effects of temperature on P450tol catalyzed hydroxylation reaction^[a]^

| Entry | T (^o^C) | Yield (%) ^[b]^ | *ee* (%) ^[b]^ |
| --- | --- | --- | --- |
| 1 | 5 | 22.0±0.6 | 96.0±0.1 |
| 2 | 10 | 43.0±0.7 | 96.0±0.1 |
| 3 | 15 | 69.0±0.8 | 96.1±0.2 |
| 4 | 20 | 69.4±0.9 | 96.0±0.1 |
| 5 | 25 | 67.6±0.4 | 95.8±0.1 |
| 6 | 30 | 62.9±0.6 | 95.6±0.1 |
| 7 | 35 | 57.7±0.4 | 95.0±0.1 |
| 8 | 40 | 54.4±2.6 | 95.1±0.0 |

[a] All experiments were performed three times with resting cells in 5 mL PB buffer (50 mM, pH = 8.0), cell density was 10 g cdw/L, substrate concentration was 2 mM, reaction were taken at temperaure gradient and 250 rpm for 6 h;

[b] The yield and *ee* were determined by chiral HPLC analysis (using CHIRALCEL OD-H column, hexane:*i*-PrOH = 90:10, 1.0 mL/min), all data were reported as mean ± S.D. (n = 3).

**Tables S2** Effects of pH on P450tol catalyzed hydroxylation reaction^[a]^

| Entry | pH | Yield (%) ^[b]^ | *ee* (%) ^[b]^ |
| --- | --- | --- | --- |
| 1 | 5.0 | 41.9±0.6 | 96.2±0.2 |
| 2 | 6.0 | 52.7±0.5 | 96.3±0.1 |
| 3 | 6.5 | 58.5±0.5 | 96.3±0.1 |
| 4 | 7.0 | 61.8±0.1 | 96.2±0.1 |
| 5 | 7.5 | 63.4±1.3 | 96.2±0.0 |
| 6 | 8.0 | 68.0±0.4 | 95.9±0.1 |
| 7 | 8.5 | 68.9±1.5 | 96.0±0.1 |
| 8 | 9.0 | 67.1±1.2 | 95.8±0.0 |

[a] All experiments were performed three times with resting cells in 5 mL PB buffer (50 mM, pH = 6.0 - 9.0), cell density was 10 g cdw/L, substrate concentration was 2 mM, reaction were taken at 20 ℃ and 250 rpm for 6 h;

[b] The yield and *ee* were determined by chiral HPLC analysis (using CHIRALCEL OD-H column, hexane:*i*-PrOH = 90:10, 1.0 mL/min), all data were reported as mean ± S.D. (n = 3)

**Table S3** Effects of cell density on P450tol catalyzed hydroxylation reaction^[a]^

| Entry | Resting cells concentration  (g cdw/L) | | Yield (%)^[b]^ | *ee* (%)^[b]^ |
| --- | --- | --- | --- | --- |
| 1 | | 5 | 49.2±1.9 | 95.5±0.1 |
| 2 | | 10 | 69.4±1.2 | 95.8±0.0 |
| 3 | | 15 | 67.5±2,1 | 95.7±0.0 |
| 4 | | 20 | 68.1±0.7 | 96.0±0.0 |
| 5 | | 25 | 70.1±1.9 | 96.1±0.1 |

[a] All experiments were performed three times with resting cells in 5 mL PB buffer (50 mM, pH = 8.5), different cell density, substrate concentration was 2 mM, reaction was taken at 20 ℃, 250 rpm for 2h;

[b] The yield and *ee* were determined by chiral HPLC analysis (using CHIRALCEL OD-H column, hexane:*i*-PrOH = 90:10, 1.0 mL/min), all data were reported as mean ± S.D. (n = 3).

**Figure S1** The P450tol-4 showed no catalytic activity toward alkynes **1m-o**.

**Figure S2** The second and third molecular dynamics simulations: (a, d) The fluctuation of the distance of the C7(**1f**)-O(heme), C10(**1f**)-O(heme) and C11(**1f**)-O(heme) during the MD simulations; (b, e) Distances determined between the O(heme) and H8(**1f**) (x axis) and angles formed by O(heme)−H8(**1f**)−C10(**1f**) (y axis) during the MD simulations (3 joint MD replicas). The red box indicates satisfactory conditions of active poses showing both the distance (H8(**1f**)-O(heme)) ≤ 2.7 Å and the angle (O(heme)−H8(**1f**)−C10(**1f**)) ≥ 150°. (c, f) The fluctuation of the H8(**1f**)-O(heme) and H9(**1f**)-O(heme) distances (y primary axis) and the O(heme)-H8(**1f**)-C10(**1f**) and O(heme)-H9(**1f**)-C10(**1f**) angles (y secondary axis) along the simulation time (x axis) for one of the replicas.

2. Characterization data for the products of chiral (*S*)-2.

**(*S*)-2a**

yellow oil, 48% yield, 95% *ee*, [α]_D_^25^ = -37.5 (c =0.50, CH_2_Cl_2_), The *ee* was determined by chiral HPLC (Chiralcel OD-H, Hexane/i-PrOH = 90/10, flow rate = 1.0 mL/min, λ = 254 nm, t(R) = 7.0 min, t(S) = 14.2 min). ^1^H NMR (400 MHz, CDCl_3_): δ 7.45 - 7.40 (m，4H), 7.33 - 7.28 (m, 3H), 4.76 (q, J = 6.6 Hz, 1H), 2.32 (s, 1H), 1.56 (d, J = 6.6 Hz, 3H). ^13^C NMR (100 MHz, CDCl_3_): δ 131.8, 128.5, 128.4, 122.7, 91.1, 84.1, 58.9, 24.5. HRMS(ESI-TOF) Calcd. for C_10_H_10_O [M+H]^+^: 147.0810; found: 147.0812.

**(*S*)-2b**

yellow oil, 22% yield, 99% *ee*, [α] _D_^25^ = -21.1 (c =0.50, CH_2_Cl_2_), The *ee* was determined by chiral HPLC (Chiralcel OD-H, Hexane/i-PrOH = 90/10, flow rate = 1.0 mL/min, λ = 254 nm, t(R) = 6.2 min, t(S) = 11.6 min). ^1^H NMR (400 MHz, CDCl_3_) δ 7.45 – 7.41 (m, 2H), 7.33 – 7.29 (m, 3H), 4.56 (t, J = 6.4 Hz, 1H), 1.89 – 1.77 (m, 2H), 1.08 (t, J = 7.4 Hz, 3H). ^13^C NMR (100 MHz, CDCl_3_) δ 131.8, 128.4, 122.8, 90.0, 85.0, 64.3, 31.1, 9.6. HRMS(ESI-TOF) Calcd. for C_11_H_12_O [M+H]^+^: 161.0966; found: 161.0966.

**2c**

yellow oil, 53% yield. ^1^H NMR (400 MHz, CDCl_3_) δ 7.46 – 7.42 (m, 2H), 7.35 – 7.25 (m, 3H), 4.50 (s, 1H), 2.15 (s, 1H). ^13^C NMR (100 MHz, CDCl_3_) δ 131.8, 128.4, 122.6, 87.3, 85.8, 51.7. HRMS(ESI-TOF) Calcd. for C_10_H_9_FO [M+H]^+^: 133.0653; found: 133.0648.

**(*S*)-2d**

yellow oil, 28% yield, 97% *ee*, [α]_D_^25^ = -34.7 (c =0.50, CH_2_Cl_2_), The *ee* was determined by chiral HPLC (Chiralcel OD-H, Hexane/i-PrOH = 90/10, flow rate = 1.0 mL/min, λ = 254 nm, t(R) = 6.0 min, t(S) = 7.4 min). ^1^H NMR (400 MHz, CDCl_3_): δ 7.43-7.37 (m，1H), δ 7.31-7.24（m，1H) , δ 7.09-7.01（m，1H），δ 4.78 (q, *J* = 6.6 Hz, 1H), 2.31 (s, 1H), 1.55 (d, *J* = 6.6 Hz, 3H). ^13^C NMR (100 MHz, CDCl_3_) δ 164.1, 161.6, 133.7, 130.3 (d, *J* = 8.0 Hz, 1C), 124.0 (d, *J* = 3.8 Hz, 1C), 115.6 (d, *J* = 21.0 Hz, 1C), 111.3 (d, *J* = 15.6 Hz, 1C), 96.3 (d, *J* = 3.4 Hz, 1C), 59.0 (d, *J* = 4.4 Hz, 1C), 24.3. HRMS(ESI-TOF) Calcd. for C_10_H_9_FO [M+H]^+^: 165.0716; found: 165.0714.

**(*S*)-2e**

yellow oil, 47% yield, 94% *ee*, [α]_D_^25^ = -102.5 (c =0.50, CH_2_Cl_2_), The *ee* was determined by chiral HPLC (Chiralcel OD-H, Hexane/i-PrOH = 90/10, flow rate = 1.0 mL/min, λ = 254 nm, t(R) = 5.6 min, t(S) = 6.9 min). ^1^H NMR (400 MHz, CDCl_3_) : δ 7.42 – 7.36 (m, 2H), 7.02 – 6.95(m, 2H), 4.74 (q, *J* = 6.6 Hz, 1H), 2.13 (s, 1H), 1.54 (d, *J* = 6.6 Hz, 3H). ^13^C NMR (100MHz, CDCl_3_) δ 162.6 (d, *J* = 248Hz, 1C), 133.7 (d, *J* = 8.2 Hz, 2C), 118.8 (d, *J* = 3.4 Hz, 1C), 115.7 (d, *J* = 22.1 Hz, 2C), 90.7, 83.1, 58.9, 24.5. HRMS(ESI-TOF) Calcd. for C_10_H_9_FO [M+H]^+^: 165.0716; found: 165.0711.

**(*S*)-2f**

yellow oil, 36% yield, 96% *ee*, [α]_D_^25^ = -41.0 (c =0.50, CH_2_Cl_2_), The *ee* was determined by chiral HPLC (Chiralcel OD-H, Hexane/i-PrOH = 90/10, flow rate = 1.0 mL/min, λ = 254 nm, t(R) = 6.6 min, t(S) = 14.3 min). ^1^H NMR (400 MHz, CDCl_3_) δ 7.27 – 7.22 (m, 2H), 7.19 (t, *J* = 7.5 Hz, 1H), 7.11-7.15 (m, 1H), 4.76 (d, *J* = 6.6 Hz, 1H), 2.32 (s, 3H), 2.26 (s, 1H), 1.55 (d, *J* = 6.6 Hz, 2H).  ^13^C NMR (100 MHz, CDCl_3_): δ138.1, 132.3, 129.4, 128.8, 128.3, 122.5, 90.7, 84.2, 58.9, 24.5, 21.3. HRMS(ESI-TOF) Calcd. for C_11_H_12_O [M+Na]^+^: 183.0786; found: 183.0788.

**(*S*)-2g**

yellow oil, 37% yield, 97% *ee*, [α]_D_^25^ = -31.4 (c =0.50, CH_2_Cl_2_), The *ee* was determined by chiral HPLC (Chiralcel OD-H, Hexane/i-PrOH = 90/10, flow rate = 1.0 mL/min, λ = 254 nm, t(R) = 7.1, t(S) = 8.4 min). ^1^H NMR (400 MHz, CDCl_3_) δ 7.24 (dd, *J* = 5.2, 1.2 Hz, 1H), 7.19 (dd, *J* = 3.7, 1.2 Hz, 1H), 6.95 (dd, *J* = 5.2, 3.6 Hz, 1H), 4.76 (q, *J* = 6.6 Hz, 1H), 2.44 (s, 1H), 1.54 (d, *J* = 6.6 Hz, 3H). ^13^C NMR (100 MHz, CDCl_3_) δ 132.3, 127.3, 127.0, 122.6, 77.4, 94.8, 59.0, 24.2. HRMS(ESI-TOF) Calcd. for C_8_H_8_OS [M+H]^+^: 153.0374; found: 153.0375.

**(*S*)-2h**

yellow oil, 50% yield, 96% *ee*, [α]_D_^25^ = -34.3 (c =0.50, CH_2_Cl_2_), The *ee* was determined by chiral HPLC (Chiralcel OD-H, Hexane/i-PrOH = 90/10, flow rate = 1.0 mL/min, λ = 254 nm, t(R) = 7.4 min, t(S) = 9.7 min). ^1^H NMR (400 MHz, CDCl_3_) δ 7.42 (dd, *J* = 3.0, 1.1 Hz, 1H), 7.24 (dd, *J* = 5.0, 3.0 Hz, 1H), 7.14 – 7.06 (m, 1H), 4.73 (q, *J* = 6.6 Hz, 1H), 2.39 (s, 1H), 1.53 (d, *J* = 6.6 Hz, 3H). ^13^C NMR (100 MHz, CDCl_3_) δ 129.9, 129.0, 125.4, 121.7, 90.7, 79.3, 58.9, 24.4. HRMS(ESI-TOF) Calcd. for C_8_H_8_OS [M+H]^+^: 153.0374; found: 153.0369.

**(*S*)-2i**

brown oil, 65% yield, 98% *ee*, [α]_D_^25^ =-42.9 (c =0.50, CH_2_Cl_2_), The *ee* was determined by chiral HPLC (Chiralcel AD-H, Hexane/i-PrOH = 90/10, flow rate = 1.0 mL/min, λ = 254 nm, t(R) = 9.2 min, t(S) = 10.1 min). ^1^H NMR (400 MHz, CDCl_3_) δ 8.73 (dd, *J* = 2.2, 0.9 Hz, 1H), 8.47 (dd, *J* = 4.9, 1.7 Hz, 1H), 7.69 (dt, *J* = 7.9, 2.0 Hz, 1H), 7.21-7.25 (m, 1H), 4.74 (q, *J* = 6.6 Hz, 1H), 1.53 (d, *J* = 6.6 Hz, 3H). ^13^C NMR (100 MHz, CDCl_3_) δ 152.0, 148.2, 139.1, 123.3, 120.4, 95.8, 80.0, 58.2, 24.2. HRMS(ESI-TOF) Calcd. for C_9_H_9_NO [M+H]^+^: 148.0762; found: 148.0755.

**(*S*)-2j**

brown oil, 44% yield, 98% *ee*, [α]_D_^25^ = -41.8 (c =0.50, CH_2_Cl_2_), The *ee* was determined by chiral HPLC (Chiralcel OD-H, Hexane/i-PrOH = 90/10, flow rate = 1.0 mL/min, λ = 254 nm, t(R) = 9.7min, t(S) = 11.5 min). ^1^H NMR (400 MHz, CDCl_3_) δ 8.74 (s, 1H), 8.52 – 8.46 (m, 1H), 7.71 (dt, *J* = 7.9, 1.9 Hz, 1H), 7.23-7.27(m, 1H), 4.75 (q, *J* = 6.6 Hz, 1H), 1.55 (d, *J* = 6.7 Hz, 3H). ^13^C NMR (100 MHz, CDCl_3_) δ 152.1, 148.3, 139.1, 123.4, 120.3, 95.7, 80.2, 58.3, 24.3. HRMS(ESI-TOF) Calcd. for C_9_H_9_NO [M+H]^+^: 148.0762; found: 148.0760.

3. Amino acid and DNA sequences of P450tol

DNA sequence of P450tol

ATGACCACCGTTGAATCTAACACCACCGCGGCGATCCCGGATGAAATCGCGCGTCAGATCGTTCTGCCGGAAGGCCACAAAGATAACGTCCCGCTGTTCGAAGCGTACCGTTGGCTGCGTGAAAACCAGCCGCTGGGTCAGGCGCGTGTTGAAGGCTACGATCCGCTGTGGCTGATCACCAAATACGCGGATCTGATGGAAGTTGAACGTCAGCCGCAGATCTTCGCGGCGGGCGGCGGCGAAGATAAAGGTAGCAACAACCCGATCCTGGCGAACCAGGCGGGTGATGAATTCACCCGCCAGCTGCTGGGCGGTAACCTGCGTATCCTGGATGCACTGCCGTACCTGGATCAGCCGGAACACAGCGTTGTTAAAGATGTTGCGTTCGATTGGTTCCGTCCGGCTAACCTGAAAAAATGGGAAGATCGCATCCGTGAAACCGCGCGTGCATCTATCGATCGTCTGCTGGCGGGTGGCCCGGATCTGGACGCGGTTCAGGAATTCGCGGTTTTCTTCCCGCTGCGTGTTATCATGAGCCTGTTCGGCGTTCCGGAAGAAGATGAACCGCGTATGATGGCTCTGACCCAGGACTTCTTCGGTGTTGCGGATCCGGATGCTCAGCGTGATGACATCGAAGCGCTGAGCCCGGATGCAGCGGCACAGCAGTGGGCGGCTACCATCGCGGACTTCTATGCTTACTTCGATGTTCTGGTTGAATCTCGTCGCGCTGAACCGCGTGATGATCTGGCTACCCTGATCGCAGTTGCTAAAGATGAAAACGGTGAATACTTCCCGAAAACCTTCGCTTATGGTTGGTTCGTTGCTATCGCGACCGCAGGCCACGATACCACCGCATCCACCCTGGCGGGTTGTCTGCAGAGCCTGGCGGCGCACCCGGAAGTTCTGGATCGTGTTAAAGGTGATCCGGATCTGATCCCGGATCTGGTTAACGAATCTCTGCGTATCGTTAGCCCGGTTAAACACTTCACCCGTGTGGCGCTGCAGGATTACGAAATGCGTGGCCAGAAAATCAAAGCGGGTGATCGTCTGATGCTGCTGTTCCAGAGCGGCAACCGTGATGCTGAAGTTTTCGACCGTCCGGATGATTTCGATATCGATCGTCGTCCGAACAAACACATCGCGTTCGGTTATGGTCCGCATATGTGCATCGGTCAGCACCTGGCAAAACTGGAACTGAAAGTTATGCTGCAGGAACTGCTGCCGCACCTGGAACGTGTTGAAGTTTCTGGTGAACCGAAACTGATCCAGACCAACTTCGTTGGTGGTCTGCGTAAACTGCCGGTTCACCTGACCTTCTCTTAA

Amino acid sequence of P450tol

MTTVESNTTAAIPDEIARQIVLPEGHKDNVPLFEAYRWLRENQPLGQARVEGYDPLWLITKYADLMEVERQPQIFAAGGGEDKGSNNPILANQAGDEFTRQLLGGNLRILDALPYLDQPEHSVVKDVAFDWFRPANLKKWEDRIRETARASIDRLLAGGPDLDAVQEFAVFFPLRVIMSLFGVPEEDEPRMMALTQDFFGVADPDAQRDDIEALSPDAAAQQWAATIADFYAYFDVLVESRRAEPRDDLATLIAVAKDENGEYFPKTFAYGWFVAIATAGHDTTASTLAGCLQSLAAHPEVLDRVKGDPDLIPDLVNESLRIVSPVKHFTRVALQDYEMRGQKIKAGDRLMLLFQSGNRDAEVFDRPDDFDIDRRPNKHIAFGYGPHMCIGQHLAKLELKVMLQELLPHLERVEVSGEPKLIQTNFVGGLRKLPVHLTFS*

4. HPLC spectra

**HPLC spectra of *rac*-2a**


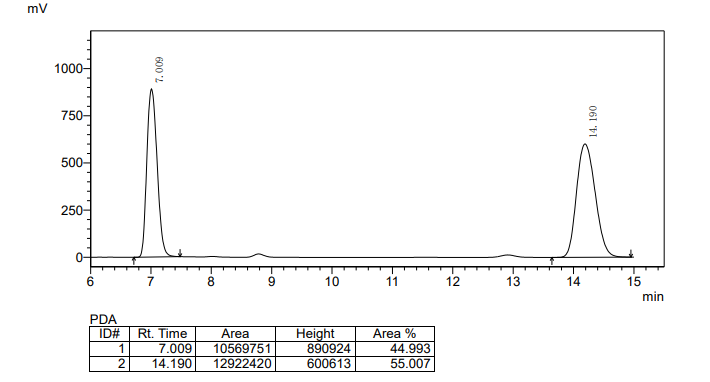


**HPLC spectra of *S*-2a**


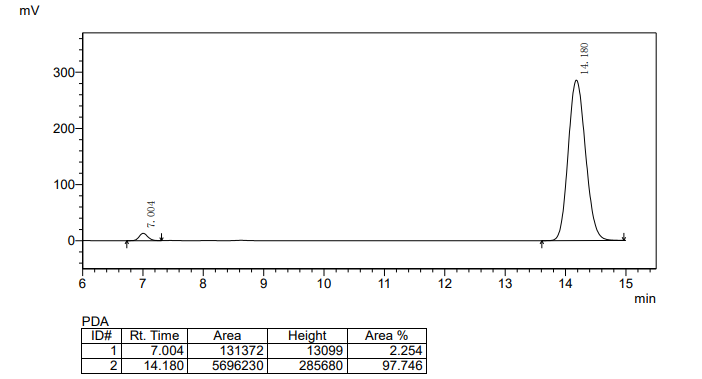


**HPLC spectra of *rac*-2b**


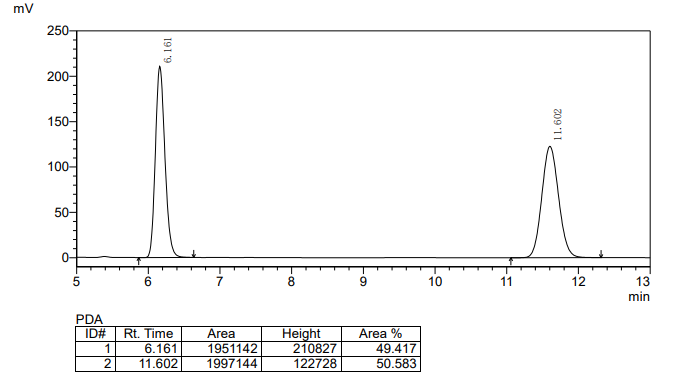

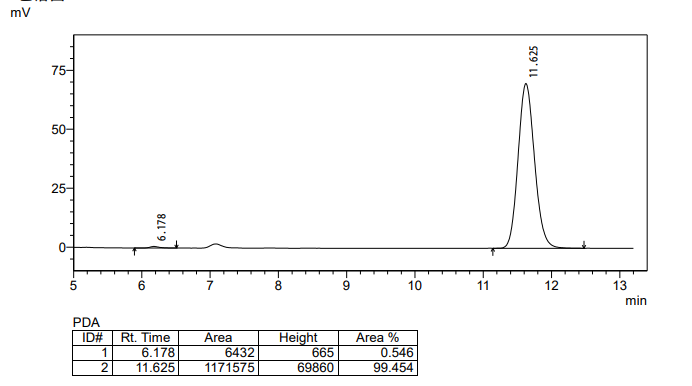


**HPLC spectra of *S*-2b**

**HPLC spectra of *rac*-2d**


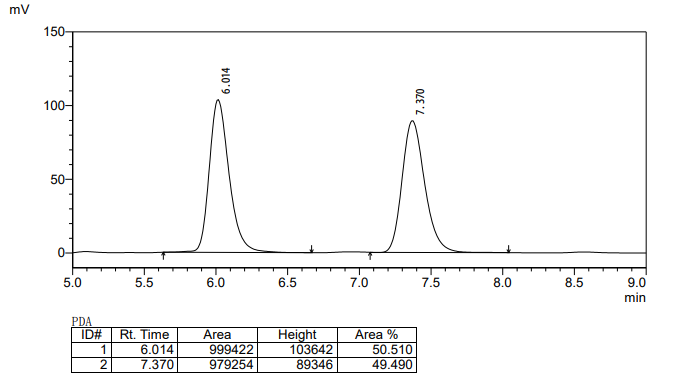

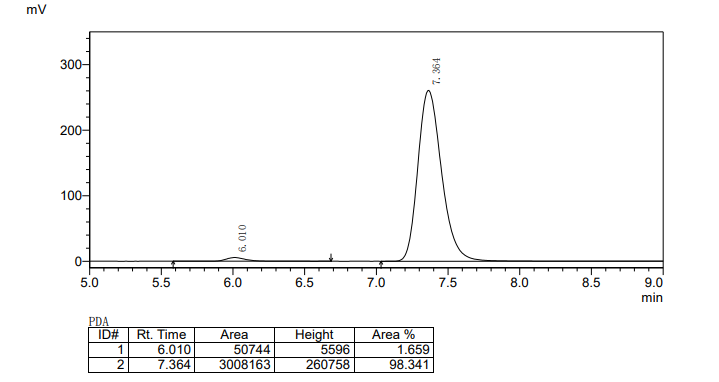


**HPLC spectra of *S*-2d**

**HPLC spectra of *rac*-2e**


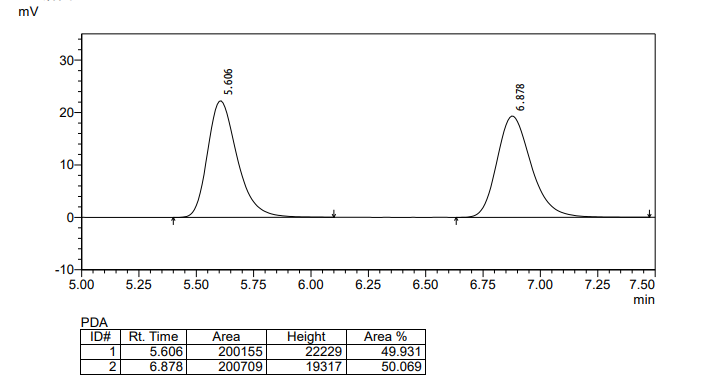

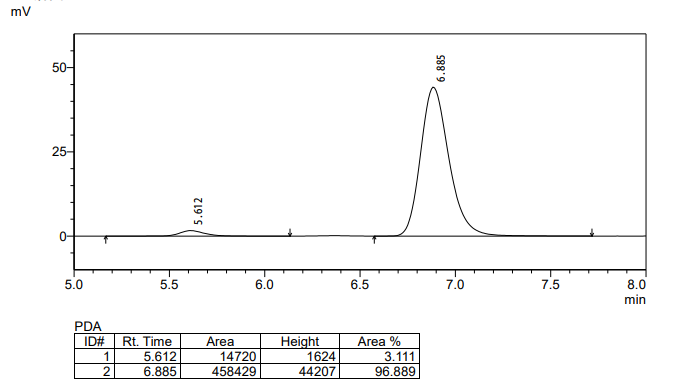


**HPLC spectra of *S*-2e**

**HPLC spectra of *rac*-2f**


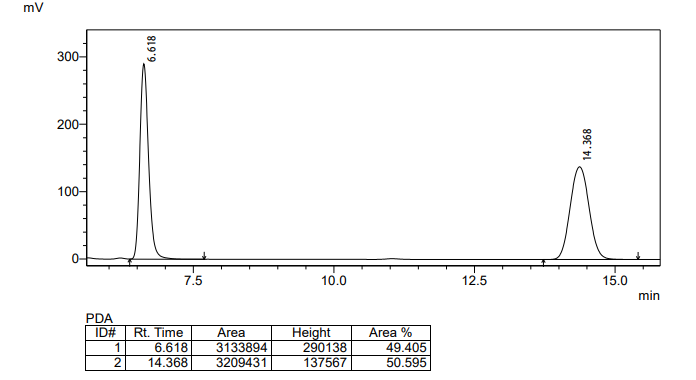

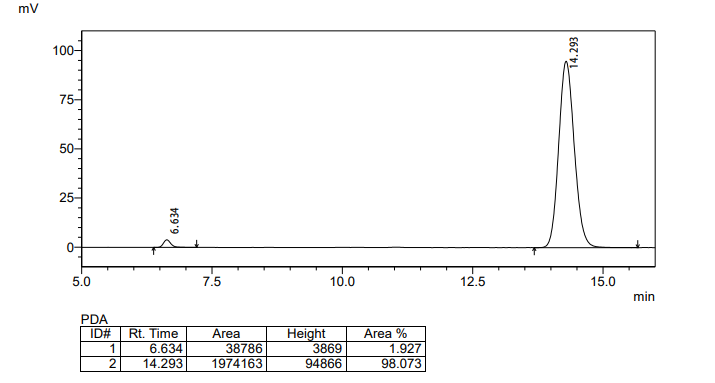


**HPLC spectra of *S*-2f**

**HPLC spectra of *rac*-2g**


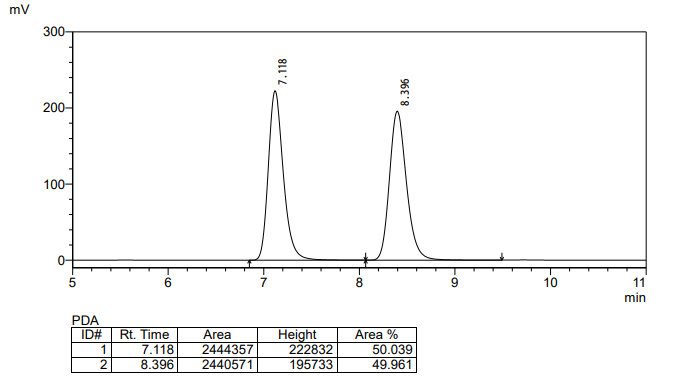

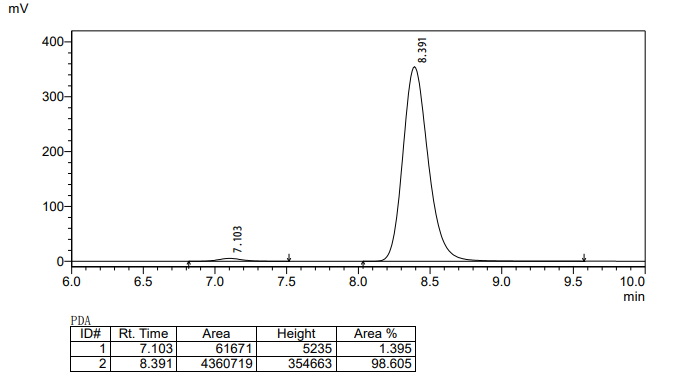


**HPLC spectra of *S*-2g**

**HPLC spectra of *rac*-2h**


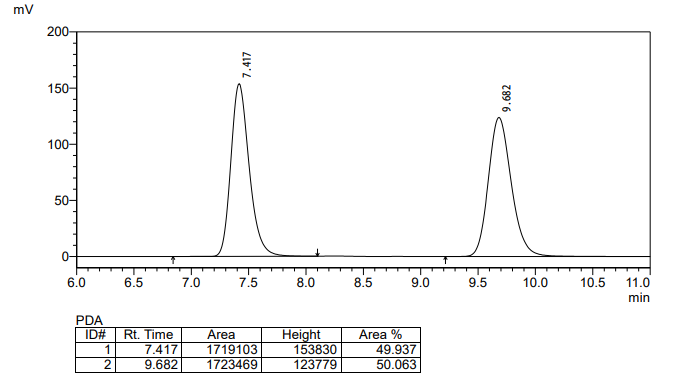

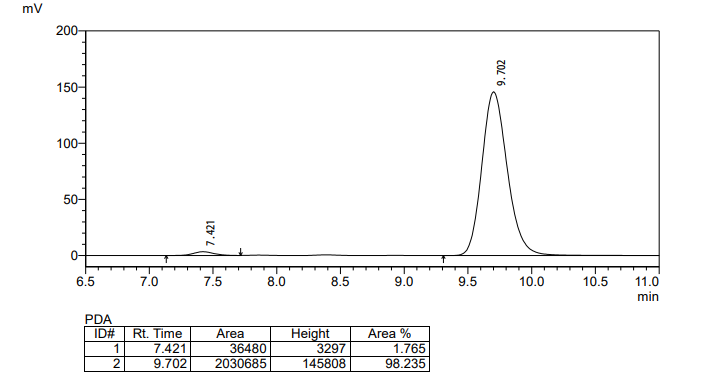


**HPLC spectra of *S-*2h**

**HPLC spectra of *rac*-2i**


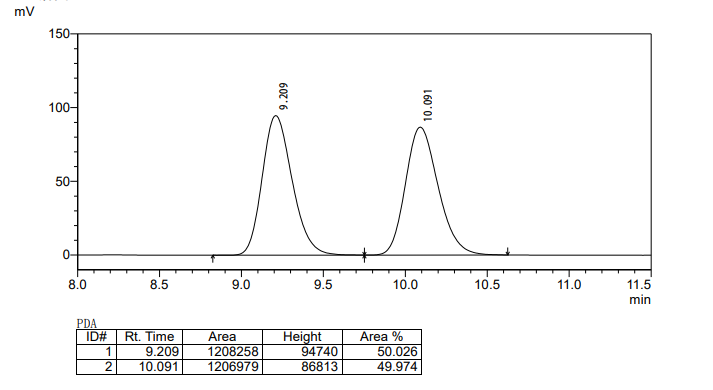

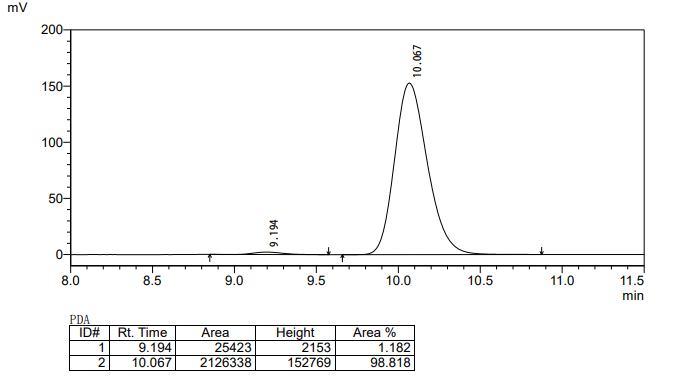


**HPLC spectra of *S-*2i**

**HPLC spectra of *rac*-2j**


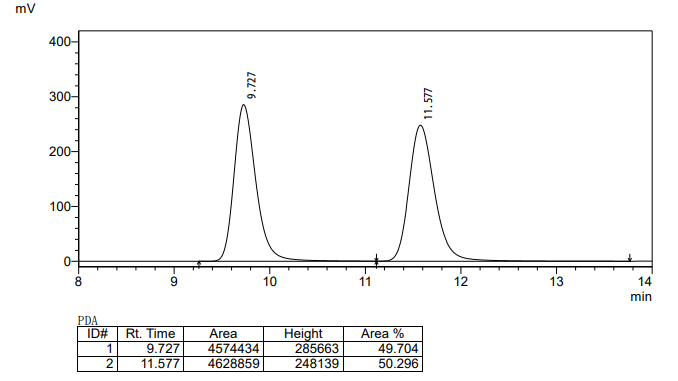

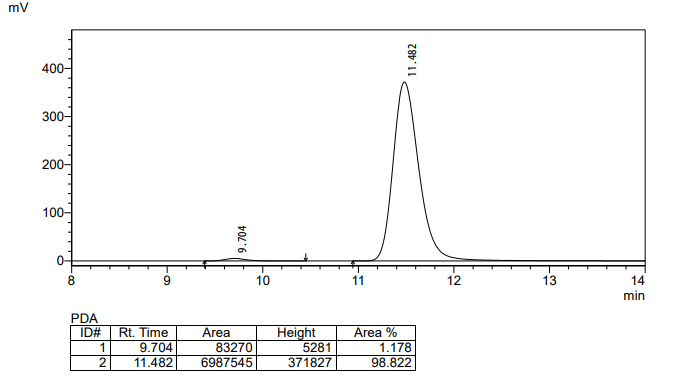


**HPLC spectra of *S*-2j**

**5.NMR spectra**

^1^H and ^13^C NMR of ***S*-2a**


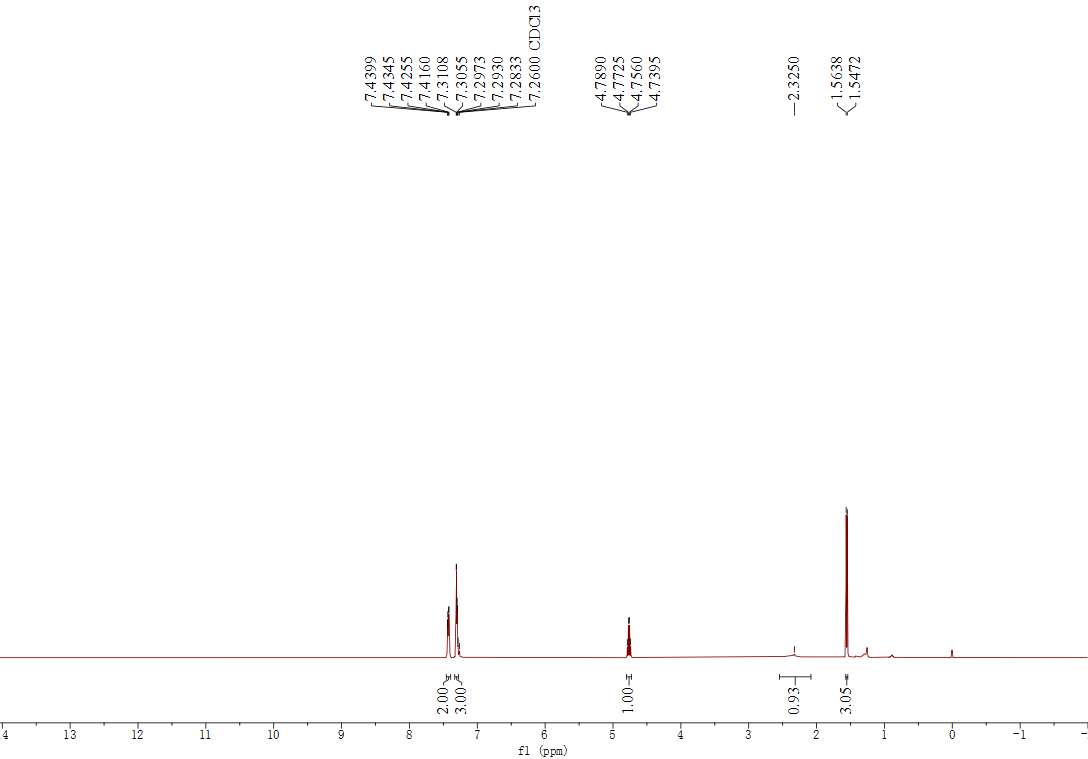

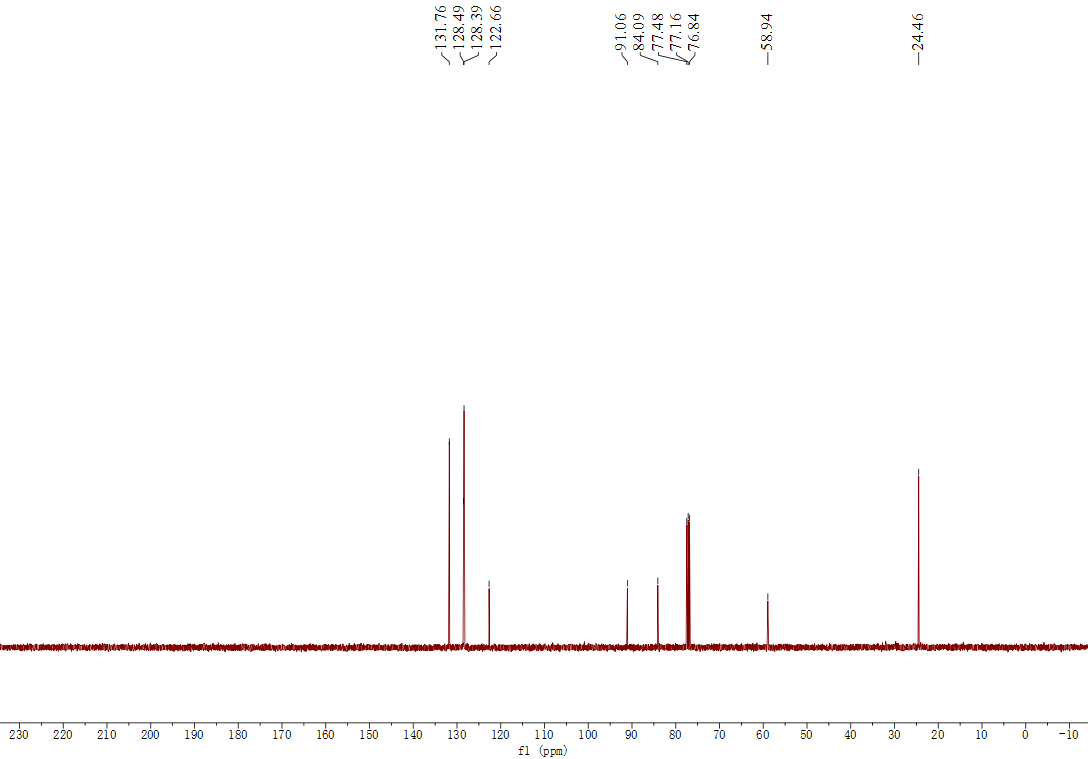


^1^H and ^13^C NMR of ***S*-2b**


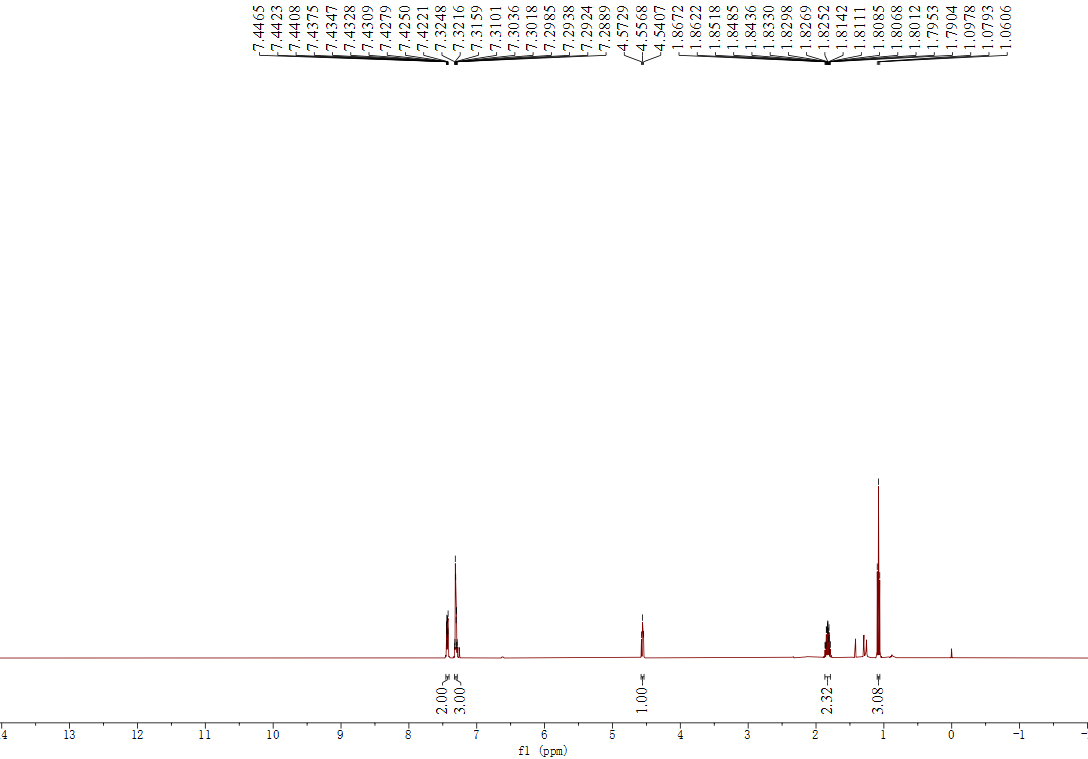

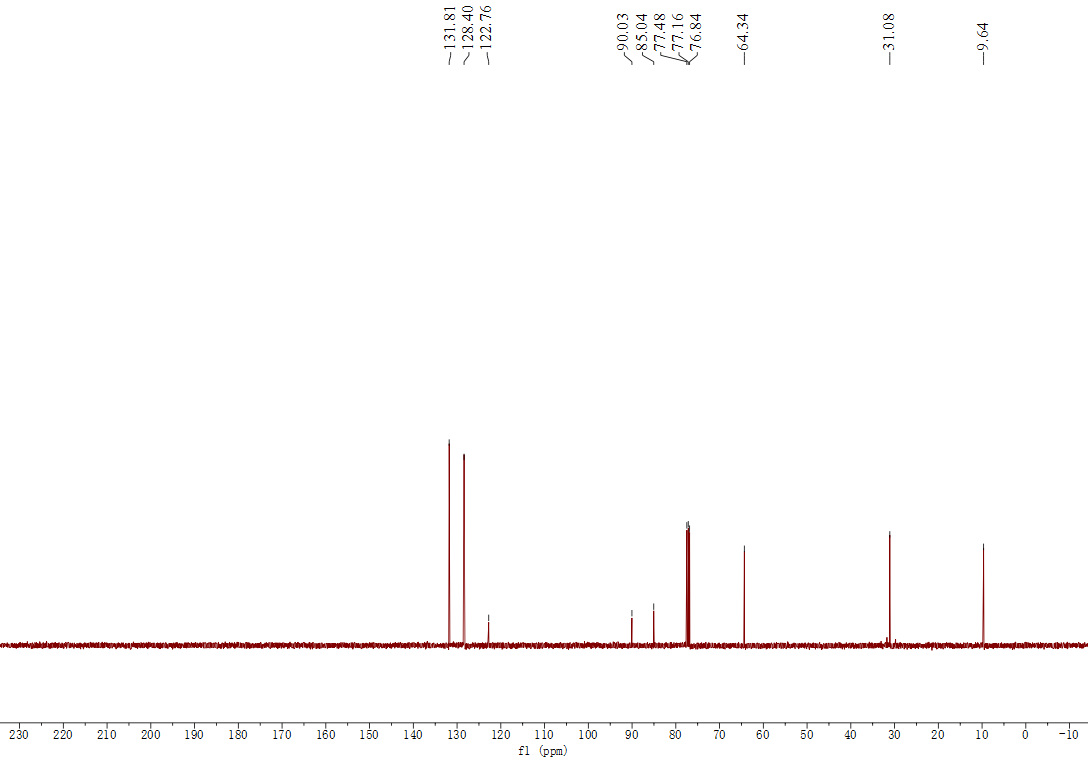


^1^H and ^13^C NMR of ***S*-2c**


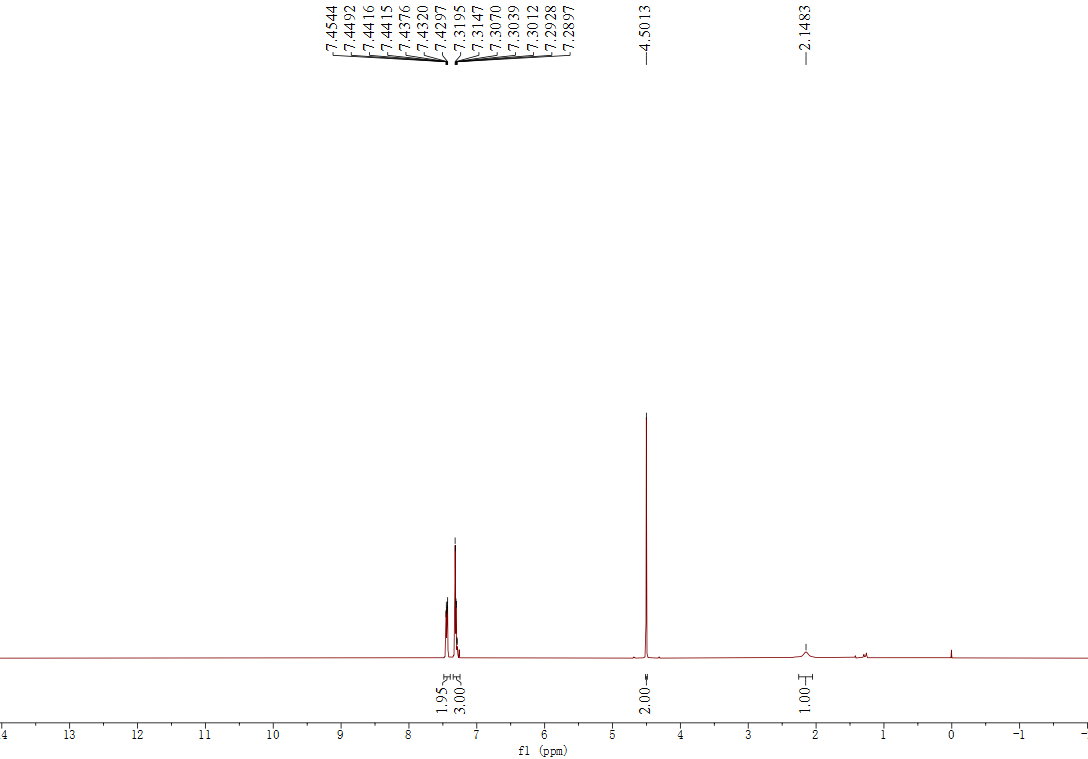

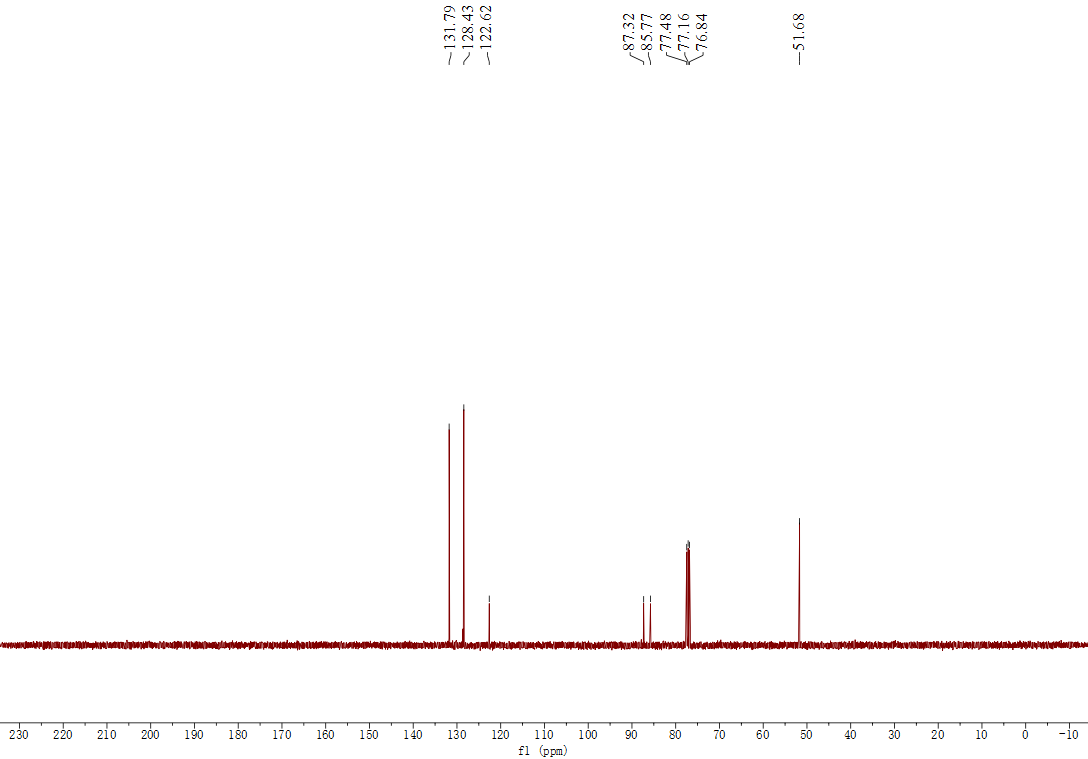


^1^H and ^13^C NMR of ***S*-2d**


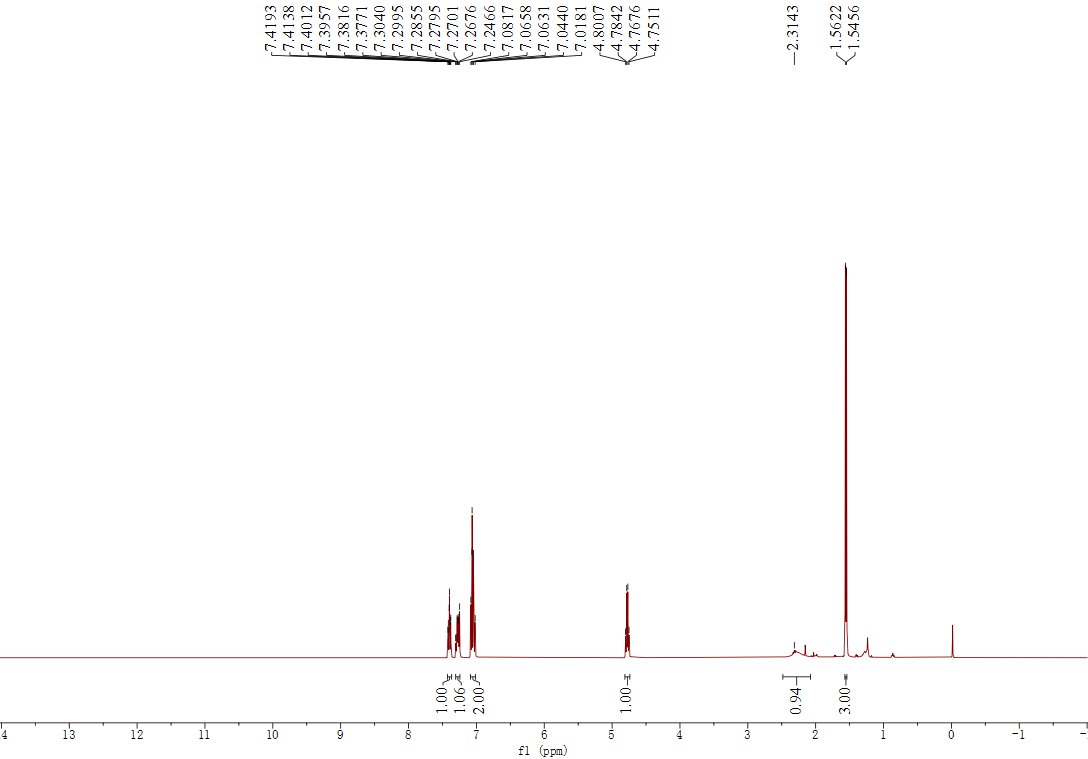

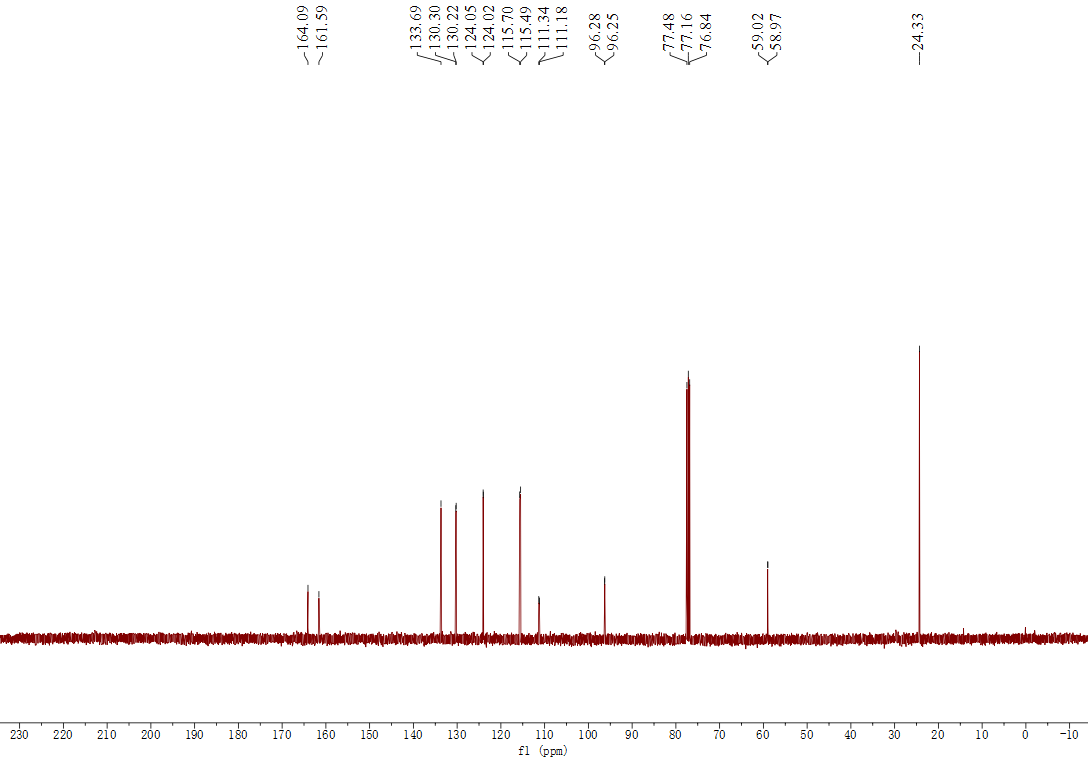


^1^H and ^13^C NMR of ***S*-2e**


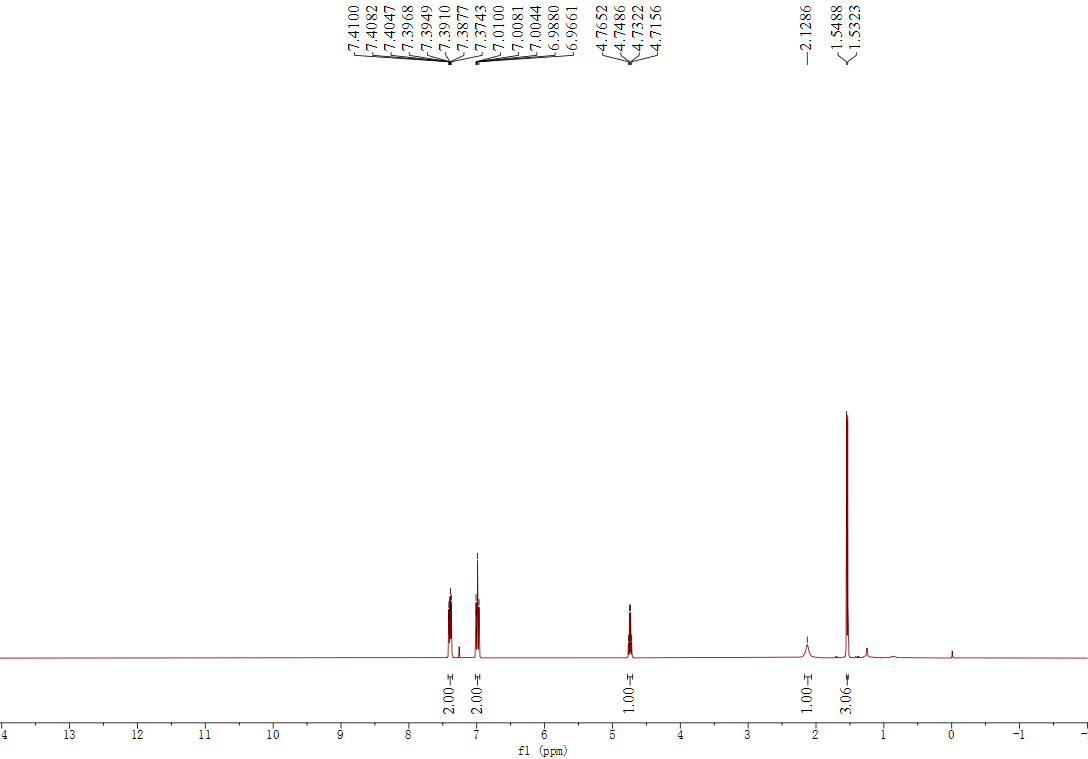

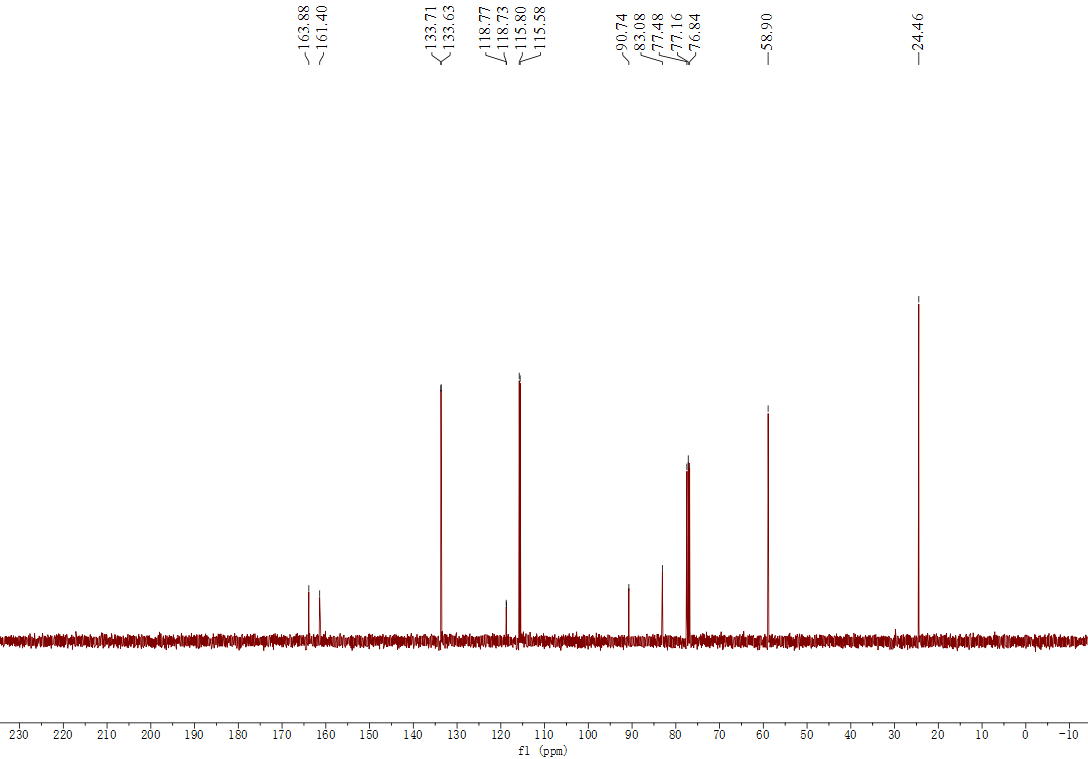


^1^H and ^13^C NMR of ***S*-2f**


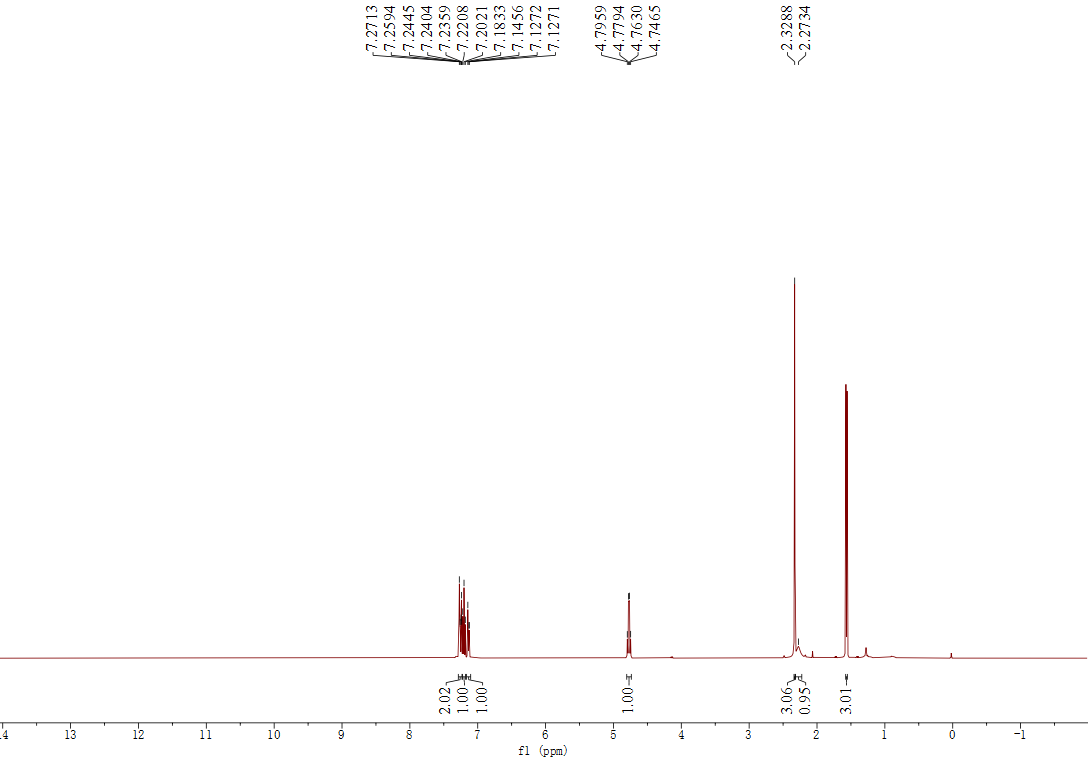

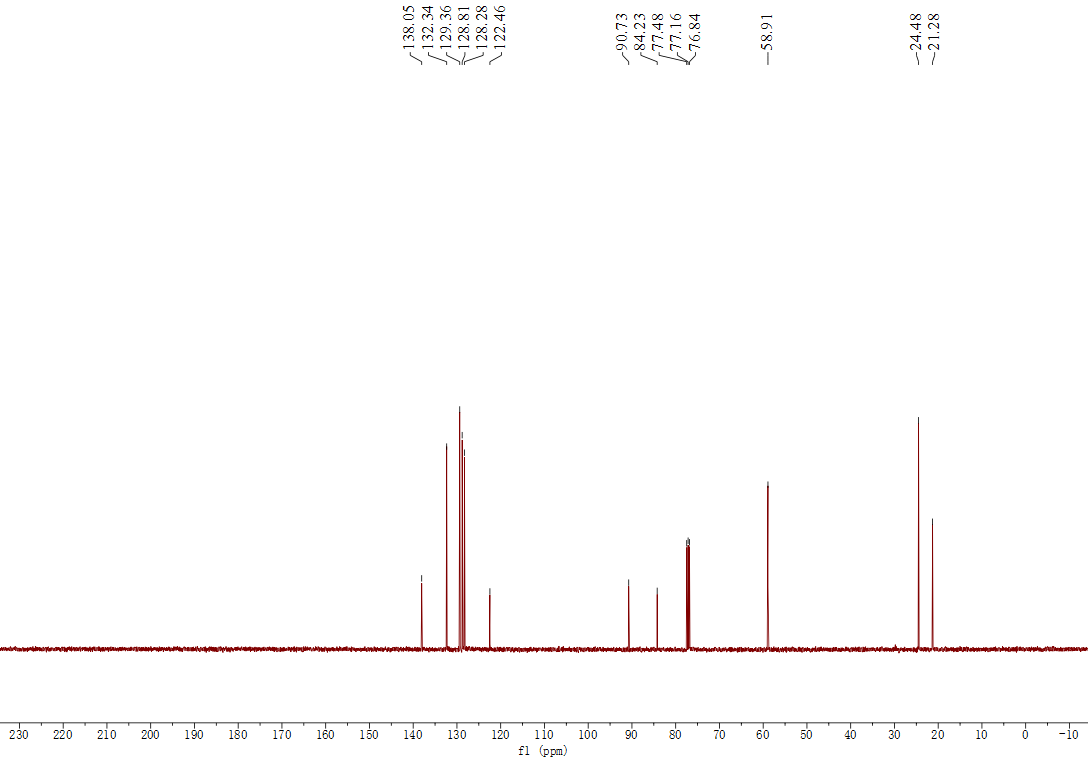


^1^H and ^13^C NMR of ***S*-2g**


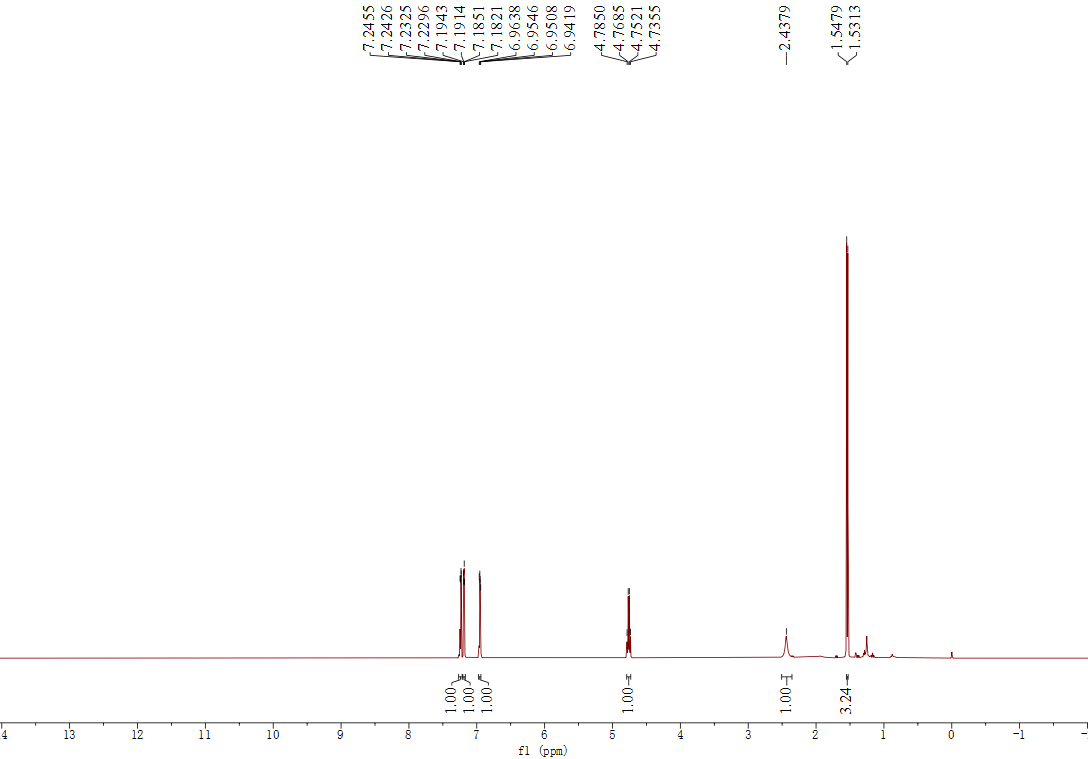

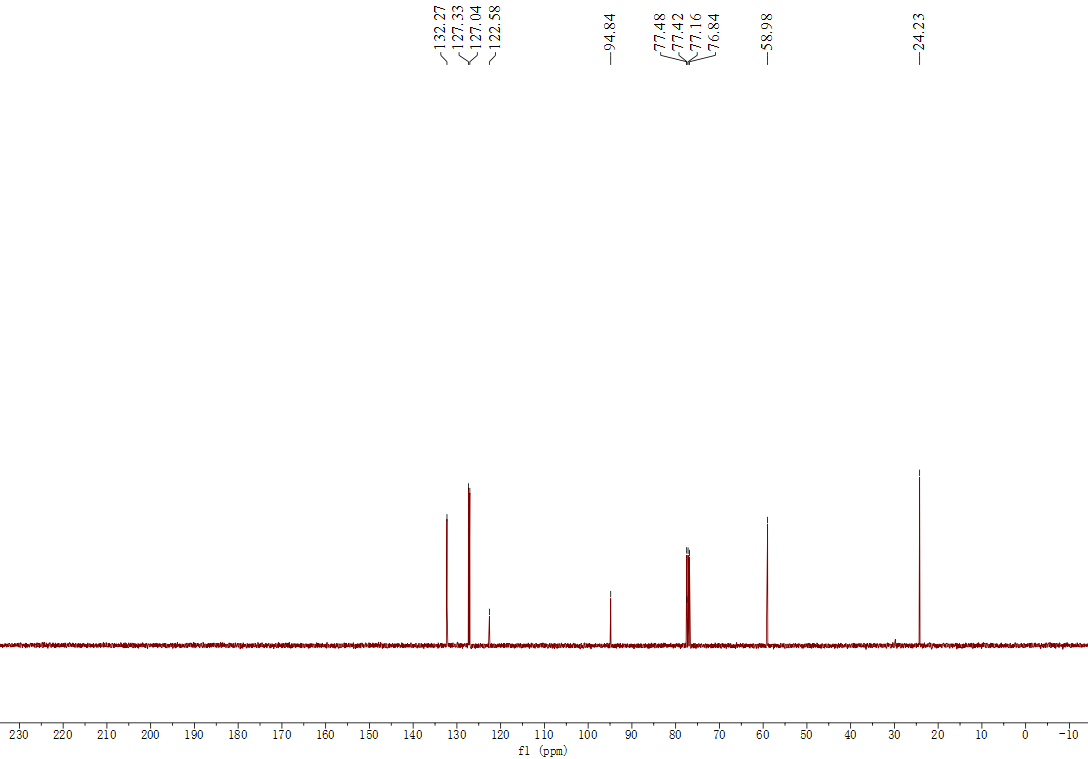


^1^H and ^13^C NMR of ***S*-2h**


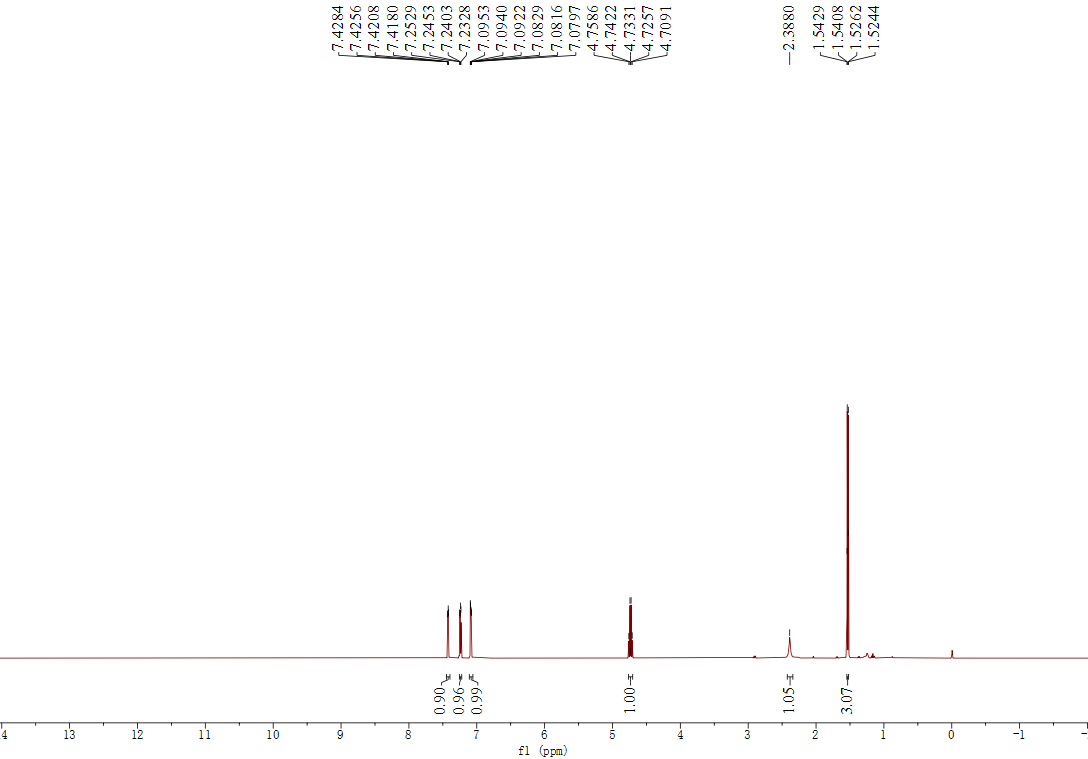

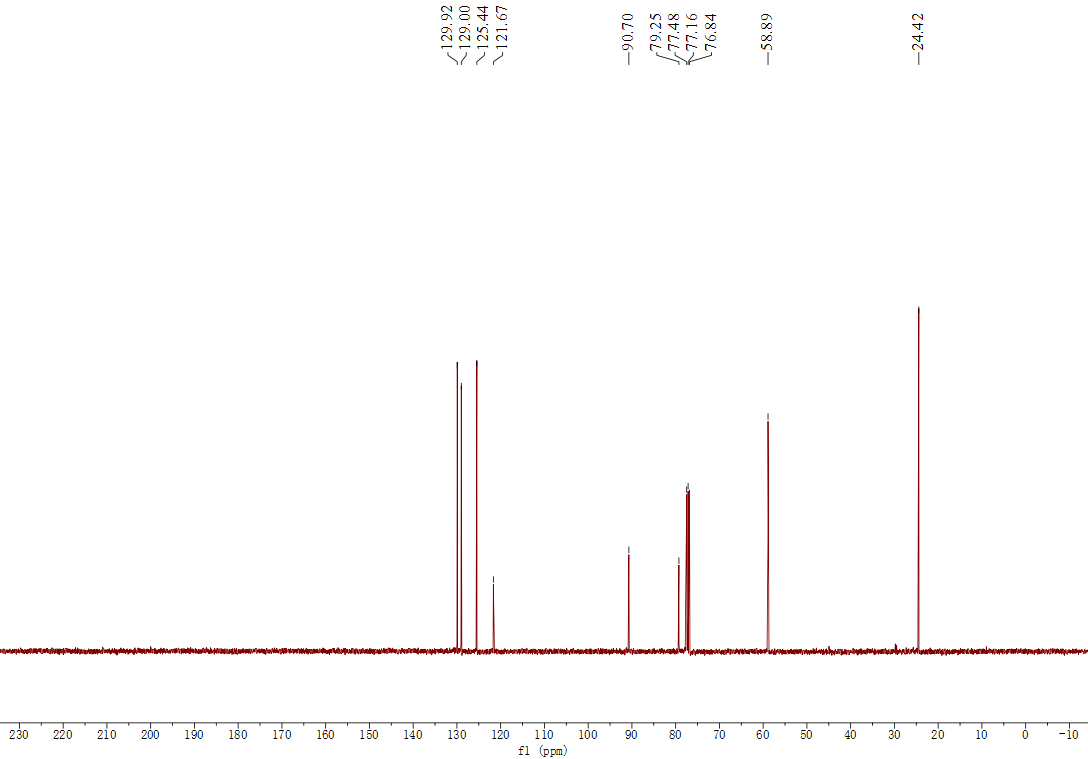


^1^H and ^13^C NMR of ***S*-2i**


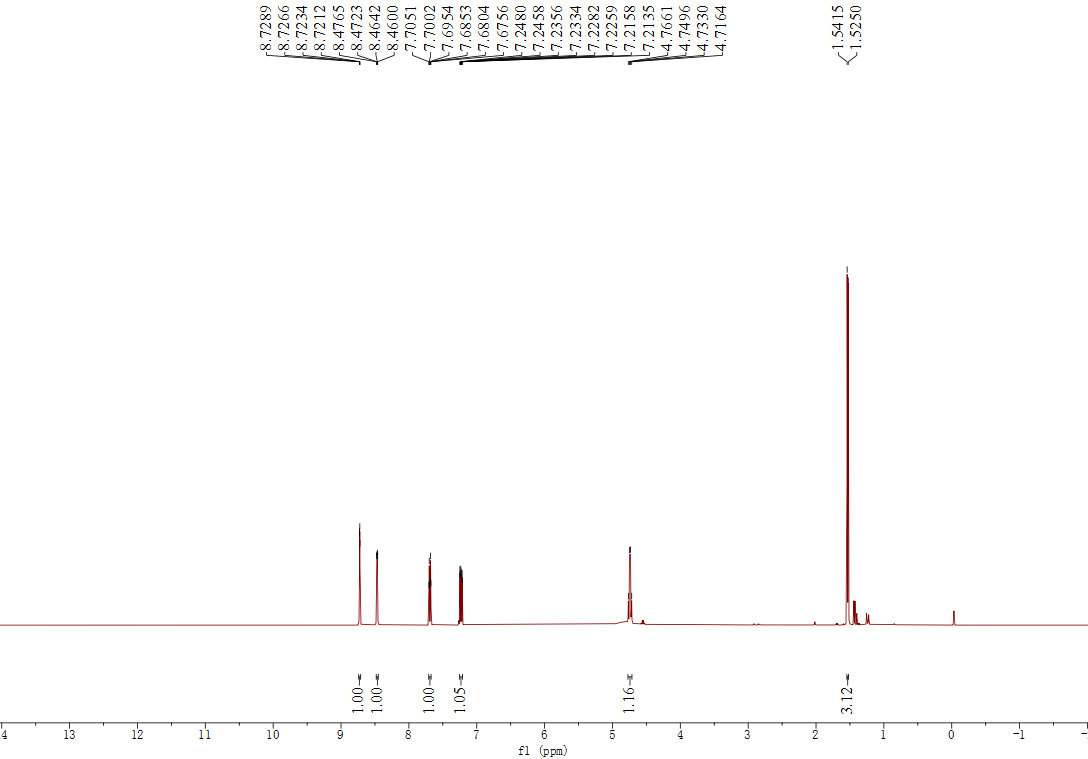

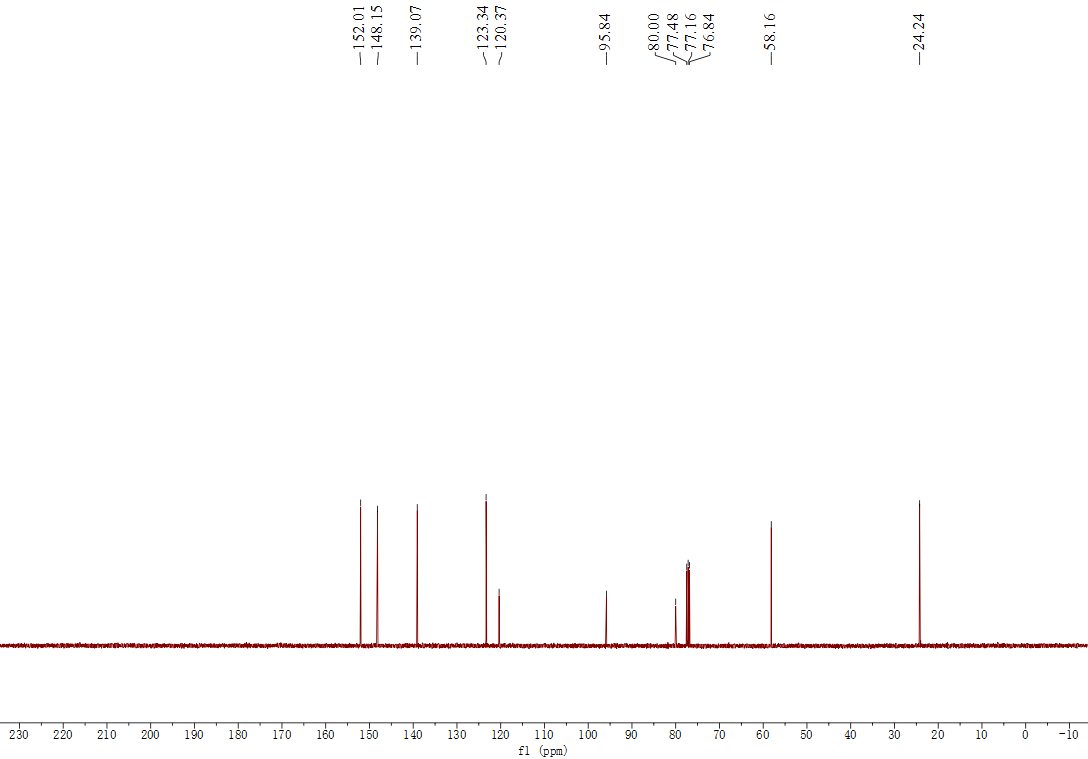


^1^H and ^13^C NMR of ***S*-2j**


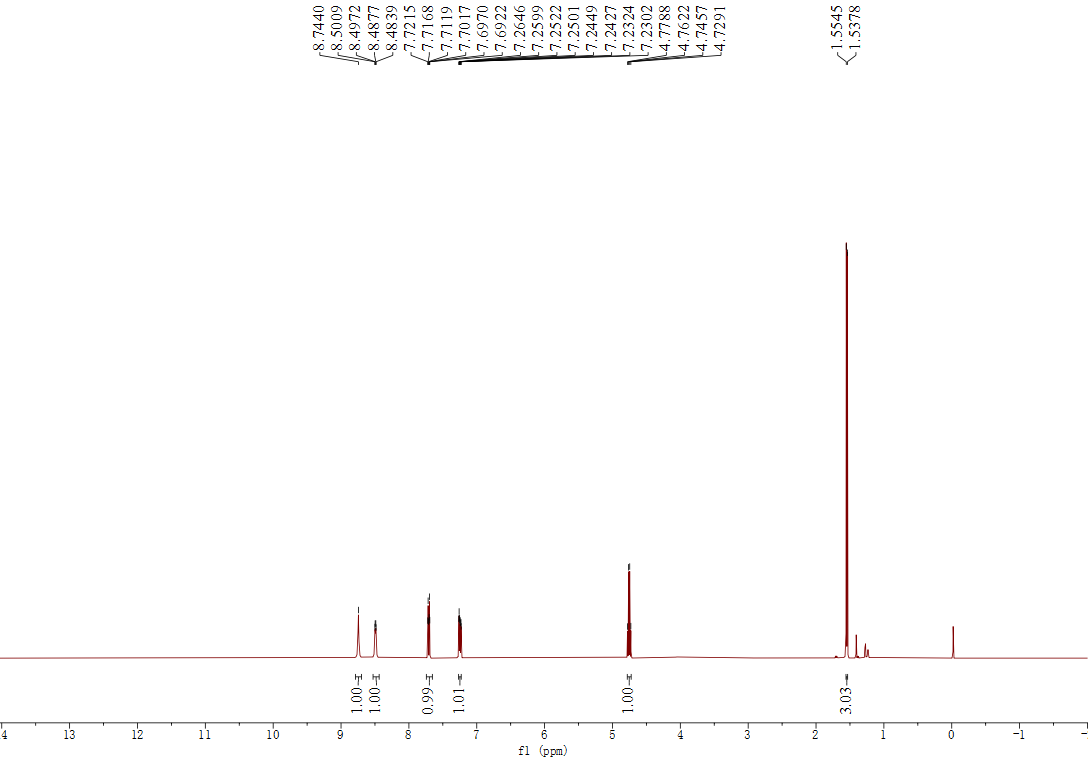

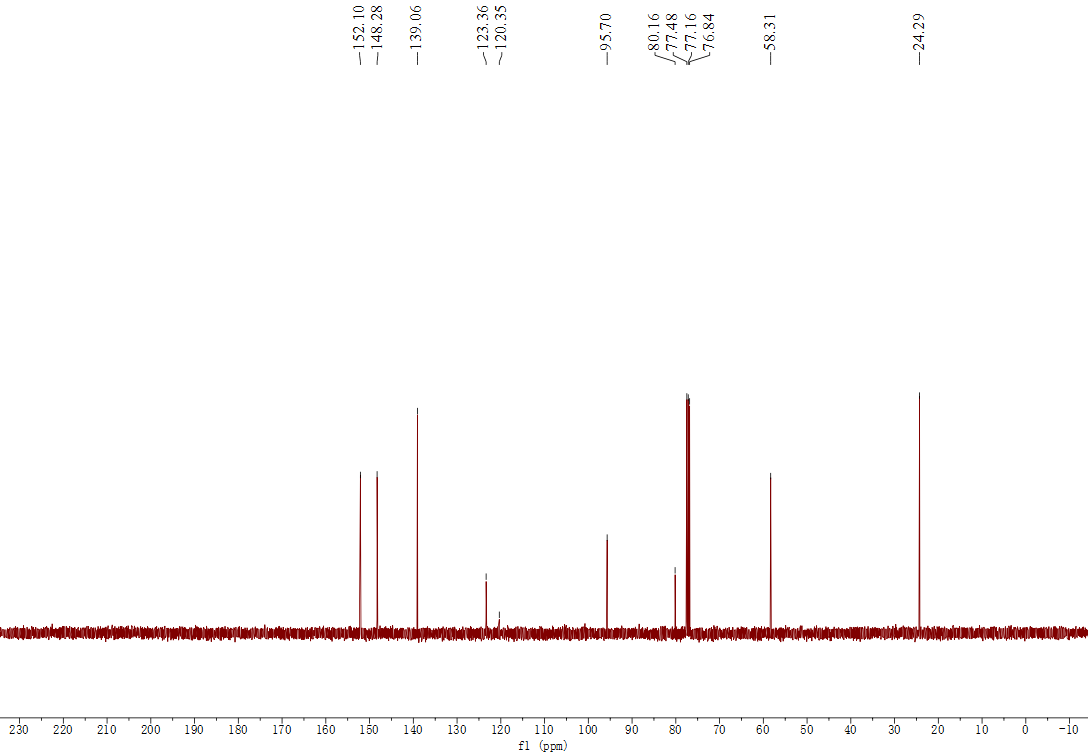

Supplement: Supplementary file 1 — Supplementary material 1: Additional Tables S1–S3, Characterization data for the products of chiral (S)-2a, Amino acid and DNA sequences of P450tol, and HPLC and NMR spectra. [file 40643_2024_771_MOESM1_ESM.doc]
